# In-Sensor-Memory Computing for Post-Von Neumann Intelligence: A Perspective

**DOI:** 10.1007/s40820-026-02191-y

**Published:** 2026-04-20

**Authors:** Hongyu Tang, Ninghai Yu, Pengsheng Min, Ruiqian Guo, Guoqi Zhang

**Affiliations:** 1 College of Intelligent Robotics and Advanced Manufacturing, Fudan University, Shanghai, 200433 People’s Republic of China; 2 Shanghai Engineering Technology Research Center for SiC Power Device, Fudan University, Shanghai, 200433 People’s Republic of China; 3 EEMCS Faculty, Delft University of Technology, Delft, 2628CD The Netherlands

**Keywords:** In-sensor-memory computing (ISMC), Post-von Neumann intelligence, Neuromorphic hardware, Industry–academia–research (IAR)

## Abstract

The rapid growth of artificial intelligence, ubiquitous sensing, and edge computing is exposing fundamental limitations of conventional von Neumann architectures, in which the physical separation of sensing, memory, and computation leads to excessive data movement, high energy consumption, and latency. As transistor scaling slows in the post-Moore era, architectural innovation has become essential to sustain progress in intelligent systems. In-sensor-memory computing (ISMC) addresses these challenges by co-locating perception, storage, and computation within unified device and system architectures, enabling in situ signal processing, mixed-signal computation, and event-driven intelligence at the data source. Recent advances in memristive and ferroelectric devices, low-dimensional and multifunctional materials, three-dimensional heterogeneous integration, and neuromorphic architectures have significantly expanded the functional scope of ISMC platforms. In parallel, the co-evolution of algorithms—including spiking neural networks, reservoir computing, and neuromorphic compilers—has facilitated the translation of device-level advantages into system-level performance. This perspective surveys the technological foundations, architectural trends, and emerging applications of ISMC, examines global industry–academia–research (IAR) collaboration, and outlines key challenges related to variability, reliability, scalability, and benchmarking. Collectively, ISMC is positioned as a post-von Neumann hardware paradigm for energy-efficient, distributed intelligence.

## Highlights

- In-sensor-memory computing (ISMC) resolves von Neumann bottlenecks via synergistic innovations across multi-dimensional functional materials, hybrid architectures, and algorithm-hardware co-design.
- This paradigm empowers ultra-low-latency edge applications, paving the way for autonomous systems, bio-integrated healthcare, and decentralized swarm intelligence.
- Translating ISMC into scalable commercialization necessitates global Industry-Academia-Research collaboration, application-centric benchmarking protocols, and cross-disciplinary ecosystem enablers.

## Graphical abstract

**Figure:**
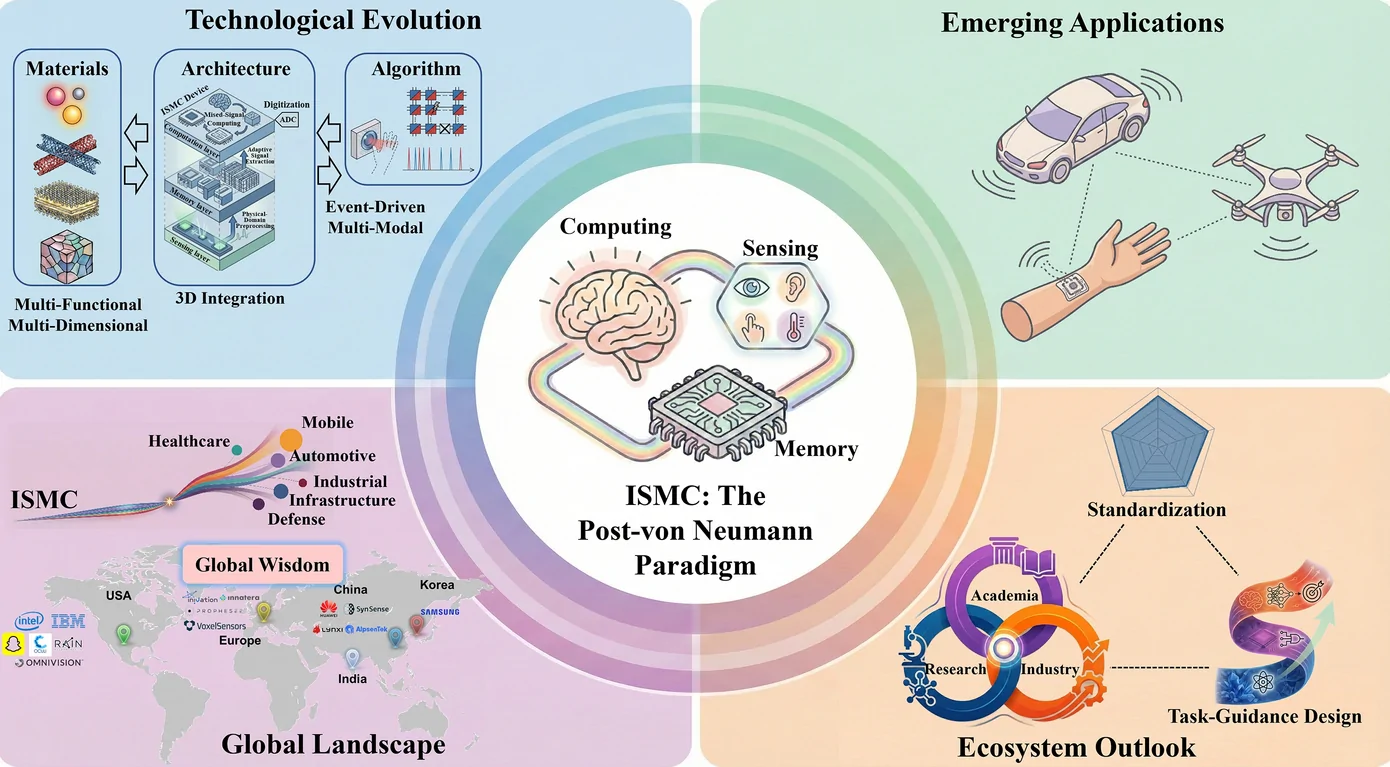

## Introduction

Over the past decade, the rapid expansion of artificial intelligence (AI), the Internet of Things (IoT), and high-bandwidth communication technologies has marked the beginning of a new wave of information innovation. As the computational backbone of intelligent manufacturing, smart cities, and emerging digital ecosystems, AI is evolving from a predominantly software-driven paradigm into a technology increasingly constrained and shaped by its hardware foundations. Although algorithms, data, and computing power are often regarded as the three pillars of AI development, recent breakthroughs in large language models (LLMs), exemplified by systems such as ChatGPT and DeepSeek, have made it evident that computing hardware has become a fundamental rate-limiting factor [1]. As model complexity and data volumes continue to grow, the performance gains achievable through conventional semiconductor scaling are no longer sufficient to meet these demands.

**Fig. 1:**
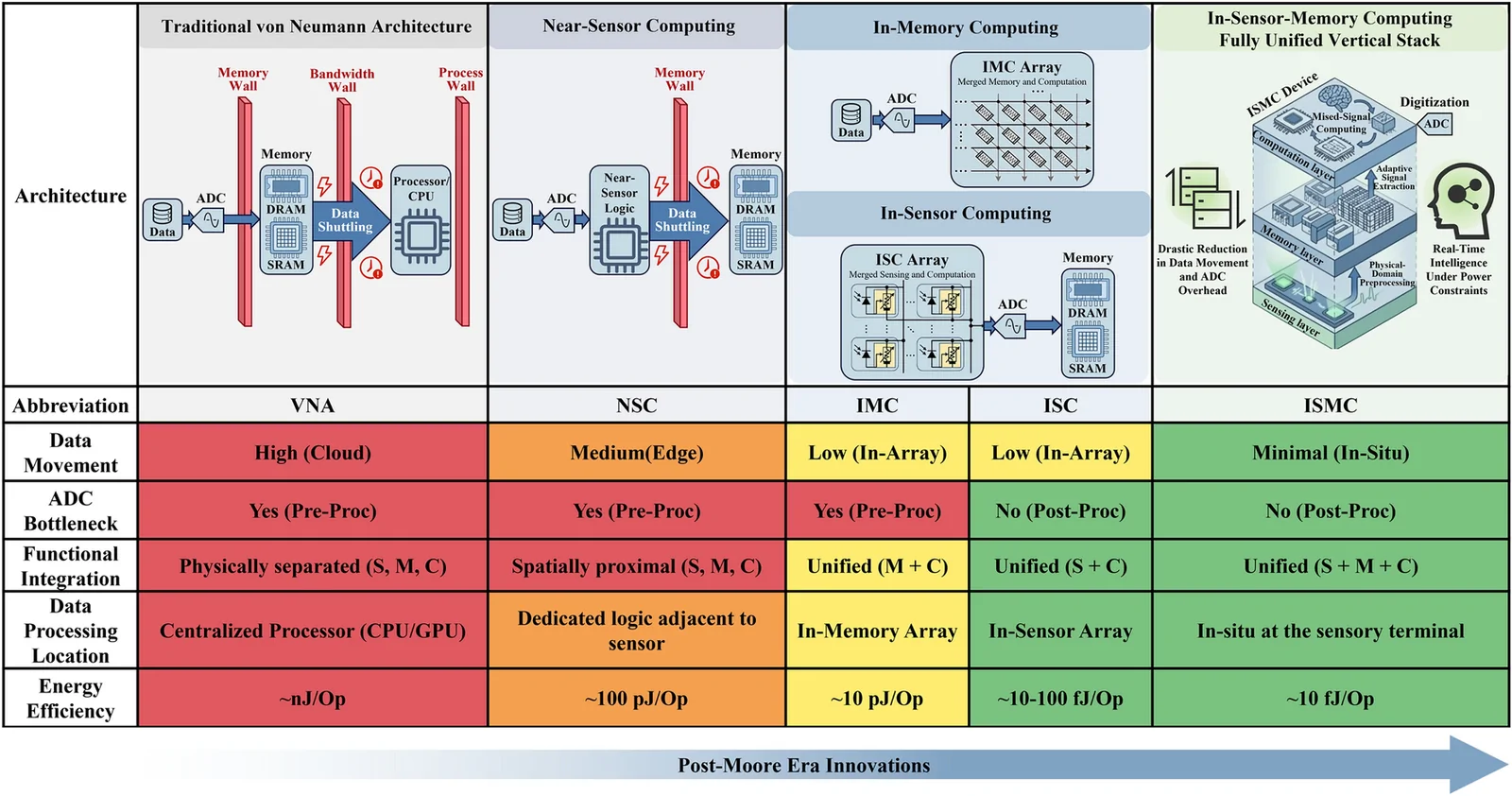
Evolution of computing architectures from traditional von Neumann to in-sensor-memory computing (ISMC). The diagram illustrates the progressive reduction in data-movement distance and energy overhead. The bottom panel quantitatively compares key metrics across architectures: data-movement scope, ADC placement relative to processing, and energy efficiency per operation. The color gradient—transitioning from red (high overhead/bottleneck) to green (minimal overhead/optimized)—signifies the increasing efficiency and integration density in the post-Moore era

This challenge signals a broader transition into the post-Moore era, in which further improvements in performance, energy efficiency, and scalability can no longer rely primarily on transistor miniaturization. Instead, fundamental limitations in device physics, power density, and fabrication cost increasingly constrain traditional technology roadmaps. At the architectural level, these pressures expose a long-standing inefficiency of von Neumann computing systems: the rigid physical separation between sensing, memory, and computation [2]. This separation leads to excessive data movement, creating well-known bottlenecks commonly referred to as the “memory wall,” “power wall,” and “bandwidth wall.” Moreover, device miniaturization is approaching its physical and economic limits, leading to the slowdown of Moore’s law and a “process wall” that constrains further performance improvements through traditional scaling [3] (Fig. 1). Here, the efficiency metrics are quantified by energy consumption per operation (pJ/Op or fJ/Op), while the placement of the analog-to-digital converter (ADC) is distinguished as either preprocessing (Pre-Proc) or post-processing (Post-Proc) relative to the computing unit. Consequently, the mismatch between AI’s escalating computational appetite and the stagnation of classical chip architectures has become one of the most fundamental challenges in modern computing.

In response, both academia and industry have accelerated efforts to explore post-von Neumann computing paradigms that reorganize how information is sensed, stored, and processed. Early approaches focused on near-sensor computing (NSC), which physically relocates digital processors closer to the sensor interface to minimize transmission latency [4]. While effective in shortening the signal path, NSC largely preserves the separation between logic and memory. Subsequently, in-memory computing (IMC) emerged as a more disruptive paradigm by physically merging memory and computation, thereby alleviating data-movement overhead and enabling massively parallel analog operations [5]. These characteristics make IMC particularly attractive for edge-intelligent systems, including wearable electronics [6], autonomous sensors [7], and IoT terminals scenarios [8], where energy efficiency outweighs traditional metrics of precision and throughput. However, a critical limitation remains shared by both paradigms: Most sensing pipelines still rely on energy-intensive ADCs and subsequent digital processing on centralized accelerators, which significantly undermines system-level efficiency. Quantitative studies show that, in resistive memory-based architectures, ADCs alone can account for up to 88% of the total readout energy [9]. Unlike IMC, which typically processes data after digitization, in-sensor computing (ISC) integrates computing elements directly into the pixel or sensor array. This allows for analog-domain processing before the signal is converted, effectively shifting the ADC from a front-end bottleneck to a backend operation. While ISC mitigates readout overheads, true system-level efficiency requires the holistic approach of in-sensor-memory computing (ISMC) [10]. By embedding memory and computation units within or directly adjacent to the sensing layer, ISMC enables physical-domain preprocessing, adaptive feature extraction, and mixed-signal computation prior to digitization. By minimizing redundant data movement and mitigating ADC overhead, ISMC represents a natural architectural evolution toward real-time, energy-efficient intelligence in the post-Moore era.

This perspective aims to articulate the technological trajectory, materials and device innovations, and system-level opportunities that position ISMC as a promising candidate for next-generation intelligent hardware. We further examine the architectural trends, algorithm-hardware co-evolution, and global industry–academia–research (IAR) dynamics shaping this emerging field and discuss the challenges that need to be addressed for ISMC to transition from laboratory demonstrations to scalable deployment.

## In-Sensor-Memory Computing (ISMC)

### Hardware Foundations of ISMC

**Fig. 2:**
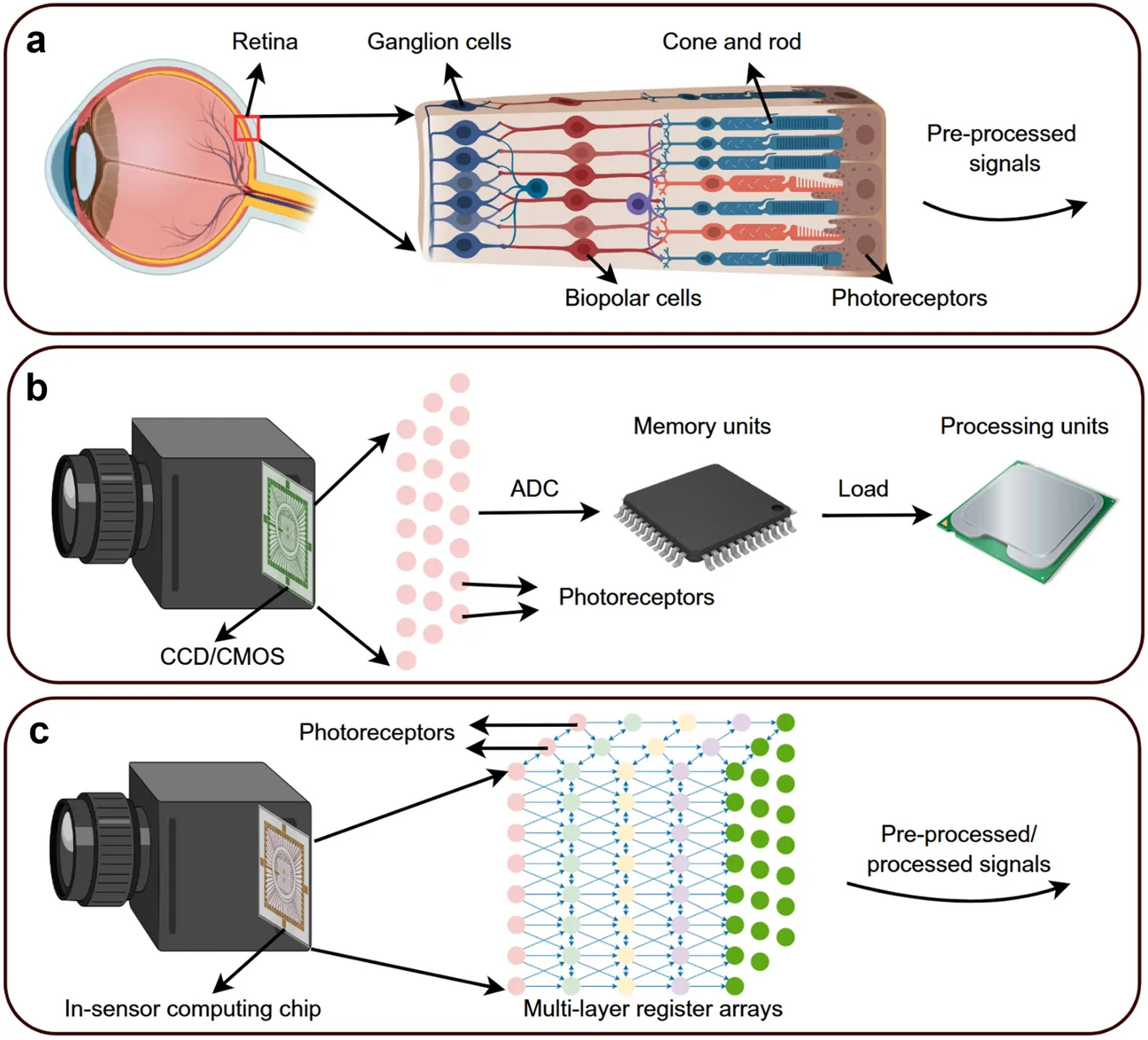
Comparison of **a** biological vision, **b** conventional sensing, and **c** ISMC architectures in brain-inspired visual perception. Reproduced from Ref. [15] with permission from Intelligent Computing, Copyright 2023

The hardware foundations of IMC can be traced back to early concepts of cellular memory arrays proposed by Kautz in 1969 [11] and were further shaped in the 1980s by the emergence of neuromorphic engineering. In particular, the work led by Carver Mead introduced IMC at Caltech [12] and established a design philosophy in which computation is performed directly where signals are generated, laying the conceptual groundwork for IMC and ISC. By exploiting the intrinsic physical dynamics of sensing elements or computing-memory devices intimately integrated with them, these systems enable the direct analog or mixed-signal processing of sensory inputs, embodying the principle of “sensing as computing, memory as processing” [13, 14]. As a natural evolution of IMC, ISMC aims to address the inefficiencies associated with handling massive sensory data in conventional architectures. Rather than treating sensing, memory, and computation as discrete functional modules, ISMC collapses these operations into a unified physical stack through device-architecture co-design. As conceptually illustrated in Fig. 2, this paradigm draws direct inspiration from biological systems, such as the human retina, where photoreceptors and neural layers perform parallel preprocessing before signals are transmitted to higher cortical centers. While early demonstrations of ISMC were largely inspired by visual perception, the concept has rapidly expanded to encompass multimodal sensory processing, integrating diverse physical stimuli beyond vision alone. Consequently, neuromorphic sensing elements can simultaneously capture, encode, and retain external stimuli within a single hardware substrate, shifting the design paradigm from “sending data to where computation happens” to “computing distributed with data” [16, 17]. Such data centricity is crucial for minimizing redundant signal generation and the heavy energy burden associated with inter-module communication.

**Fig. 3:**
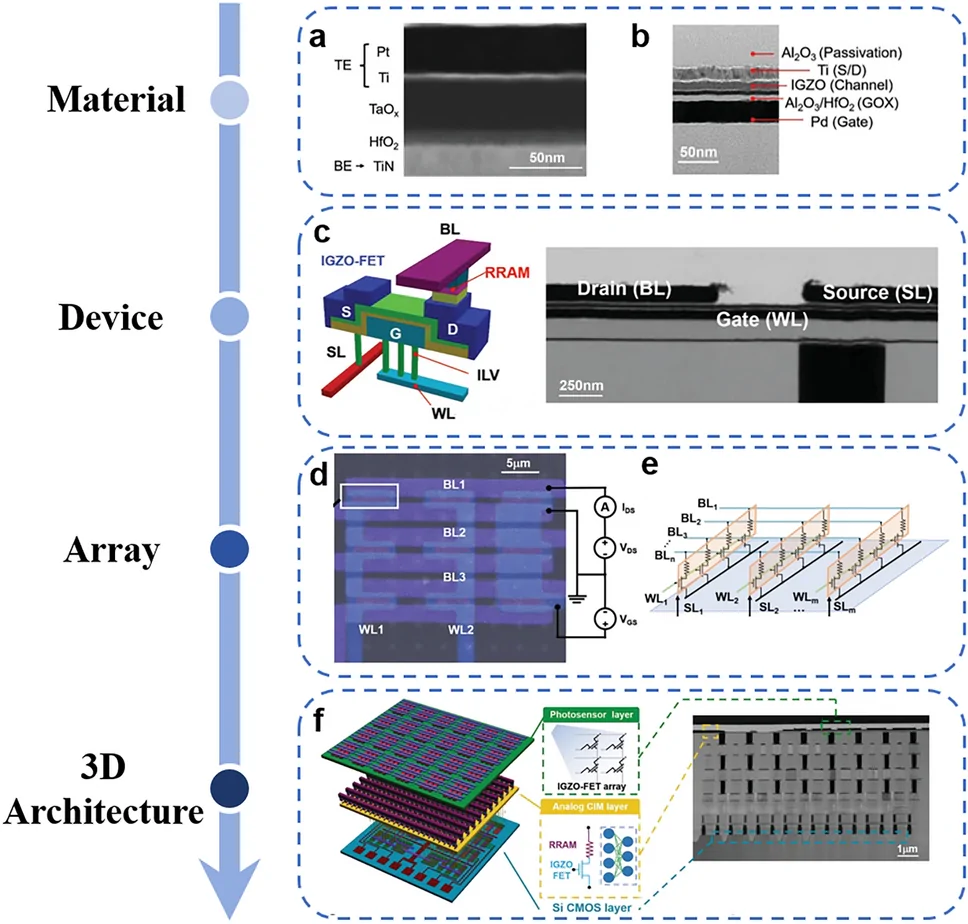
Process flow of a typical ISMC. **a** TEM image of the HfO_2_/TaO_x_ RRAM. **b** TEM image showing the material stack of the IGZO-FET. **c** 3D schematic of the 1T1R cell and TEM image of a back-gated IGZO-FET. **d** False-color SEM image of a 3 × 3 photosensor array on the chip. **e** Mapping of one convolution kernel in CNN on the IMC array. **f** 3D schematic of the M3D-SAIL chip. Reproduced from Ref. [22] with permission from Advanced Materials, Copyright 2023

Architecturally, ISMC systems adopt massively parallel and spatially distributed topologies reminiscent of biological nervous systems [18, 19]. The retina, for instance, performs edge extraction, motion detection, and contrast enhancement prior to cortical processing, providing a canonical example of ISC. Inspired by this hierarchical organization, modern ISMC platforms increasingly rely on three-dimensional (3D) heterogeneous integration to vertically stack sensing, memory, and computing layers [20, 21]. Such architectures not only shorten signal paths but also enable high-density interconnectivity and concurrent data processing across layers. Figure 3 illustrates the 3D integration fabrication flow of the monolithic three-dimensional integration (M3D-SAIL) chip, covering materials, devices, the array, and stacking [22]. The process began with the fabrication of the first-layer Si-based complementary metal–oxide–semiconductor (CMOS) control circuits on a Si substrate. Subsequently, the second-layer analog computing-in-memory array, based on a one-transistor-one-resistor (1T1R) configuration with resistive random access memory (RRAM) and an InGaZnO field effect transistor (IGZO-FET), was constructed. Key steps include: depositing a palladium (Pd) back gate and an atomic layer deposition (ALD) dielectric layer, depositing an IGZO channel layer via ALD and patterning Ti source/drain contacts, followed by depositing TiN as the RRAM bottom electrode and passivating the devices. The RRAM was then formed by depositing a hafnium oxide/tantalum oxide (HfO_2_/TaO_x_) switching layer and a Ti/Pt top electrode. Finally, the third-layer photosensor array was fabricated using a similar process, except that its IGZO channel layer was deposited by radio-frequency (RF) sputtering, and Pd was used for the source/drain contacts. This sequential integration process achieved the monolithic 3D vertical stacking of the sensor, computing core, and logic circuits.

Impressively, the process flow of ISMC, spanning material engineering, device fabrication, array integration, and 3D heterogeneous stacking based on staircase electrodes, not only meets the demands of ISMC systems for high synaptic density and energy efficiency, but also ensures manufacturability, long-term reliability, and system-level scalability through innovative thermal management, interconnect solutions, and self-rectifying mechanisms. It thereby provides a solid technological foundation for next-generation, high-efficiency artificial intelligence hardware. The vision relies heavily on emerging non-volatile memory (NVM) devices [23], including phase-change random access memory (PCRAM) [24], RRAM [25], magnetoresistive random access memory (MRAM) [24], and ferroelectric field effect transistors (FeFETs) [26, 27].

**Fig. 4:**
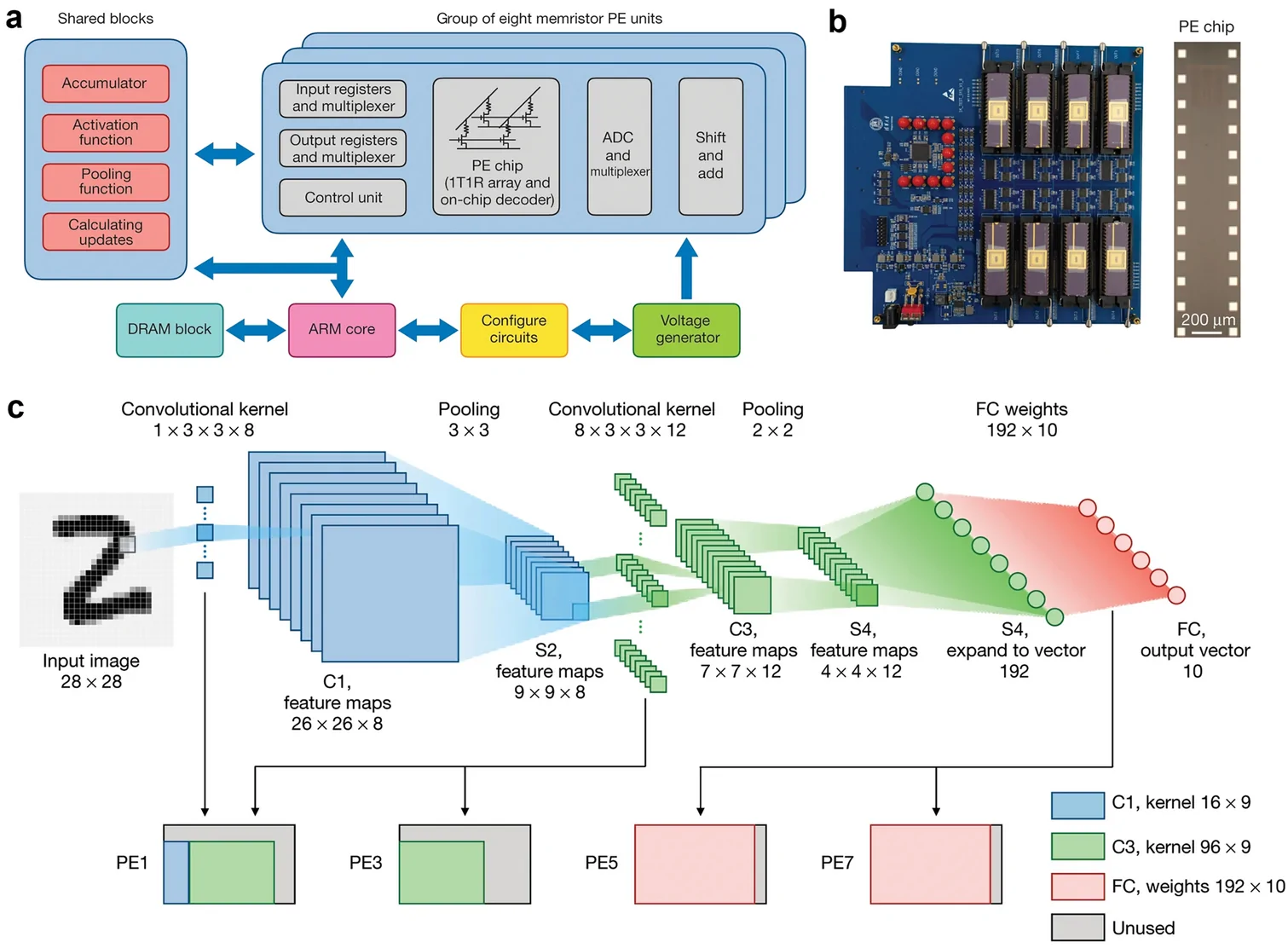
Hardware implementation of convolutional neural network (CNN) based on memristor CBAs. **a** Schematic of the system architecture with eight integrated memristor processing elements (PEs). **b** Images of PE board (left) and a partial PE chip consisting of a 2,048-memristor array and on-chip decoder circuits (right). **c** Structure of the five-layer mCNN used for MNIST image recognition. Reproduced from Ref. [29] with permission from Nature, Copyright 2020

Among emerging device platforms, memristors remain central to ISMC owing to their continuously tunable conductance states and non-volatile retention under electrical or optical stimuli [28]. These properties naturally map onto synaptic weighting and analog multiply–accumulate (MAC) operations. In particular, memristor crossbar arrays (CBAs) enable massively parallel analog computation by directly exploiting Ohm’s and Kirchhoff’s laws, thereby bypassing energy-intensive digital logic and data movement. Representative system-level demonstrations highlight the computational potential of memristor-based ISMC architectures. Yao et al. [29] experimentally demonstrated a fully hardware-implemented memristor convolutional neural network (mCNN) with hybrid training and parallel computing on multiple memristor CBAs (Fig. 4). In this scheme, input voltages applied to bitlines generate currents proportional to device conductance, while current summation along source lines yields MAC results directly in the analog domain. By eliminating repeated ADC and centralized processing, such architectures achieve high parallelism and improved energy efficiency at the hardware level.

Beyond device physics, the rapid expansion of ISMC capabilities reflects growing innovation in advanced materials, device structures, and unconventional computing mechanisms. Low-dimensional and heterostructured systems [30, 31] continue to enrich the memristor design space, while spintronic concepts [32] such as domain-wall devices [33], racetrack memory [34], and skyrmionic structures [35] offer new degrees of freedom for low-energy, high-density state manipulation. Collectively, these developments push ISMC devices toward higher functional density, lower power consumption, and progressively more adaptive behavior. Consequently, ISMC chip development is inherently multidisciplinary, requiring deep co-optimization across material science [36, 37], system architecture, heterogeneous integration [38], algorithm-hardware co-design [39, 40], and event-driven computing frameworks [41]. The broad convergence has enabled ISMC prototypes to be validated across diverse application domains, including machine vision, robotics, biosignal processing, aerospace computing, computational neuroscience, virtual reality, wearable systems, precision agriculture, and smart-city infrastructure [42, 43]. Across these scenarios, the ability of ISMC to process massive data streams at low latency and unprecedented energy efficiency highlights its potential to address the long-standing scalability and power constraints of conventional computing architectures.

### System-Level Advantages of ISMC

**Fig. 5:**
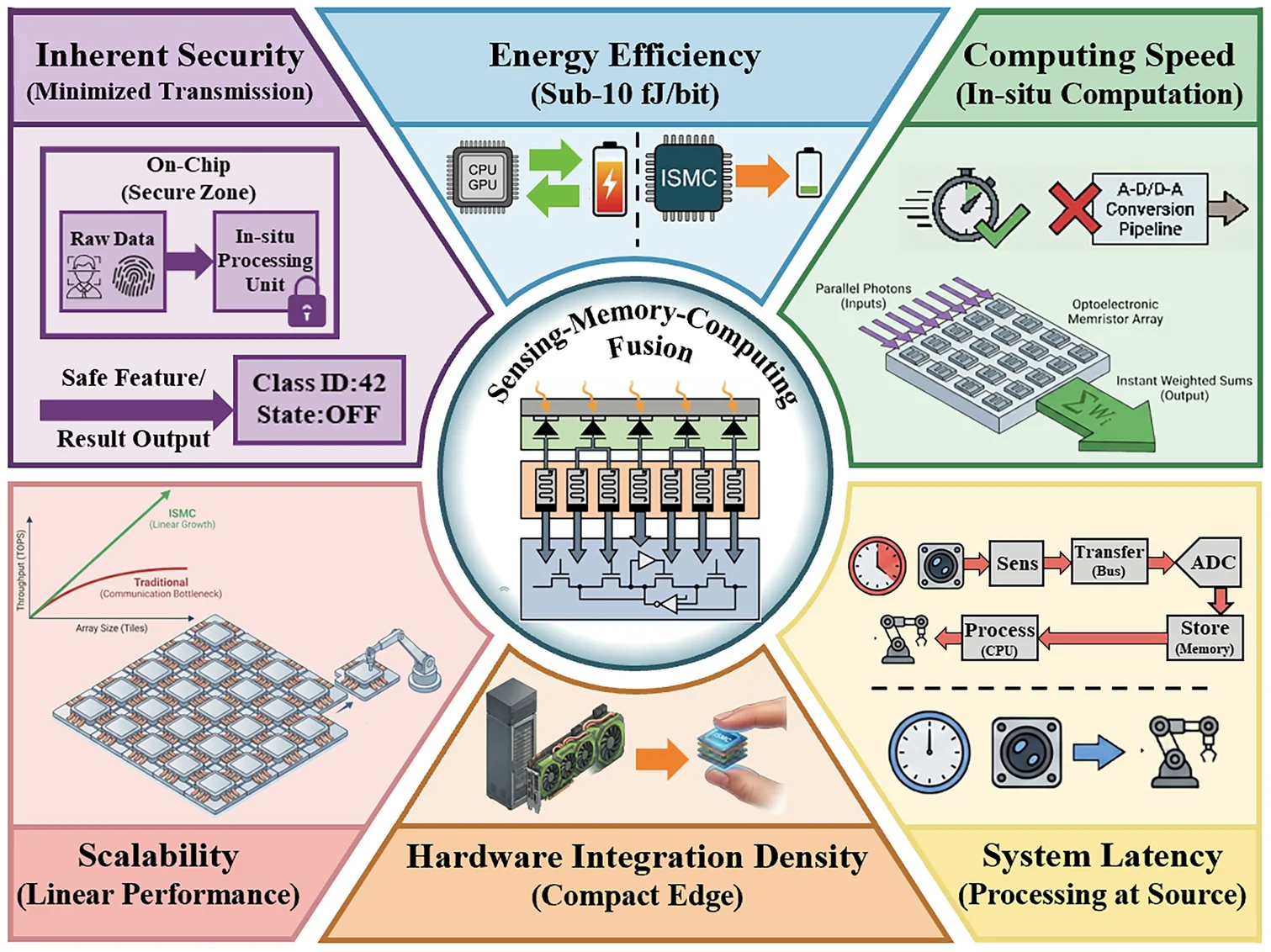
Comprehensive overview of the core technological advantages of the ISMC architecture

Conventional von Neumann architectures physically separate sensing, memory, and computation, resulting in substantial energy and latency overhead when processing large-scale sensory data. These bottlenecks originate from the massive, repetitive shuttling of data among sensors, memory hierarchies, and processors. As sensing resolutions increase and multimodal perception becomes ubiquitous, this cross-module data traffic produces high latency, saturates bandwidth resources, and dramatically increases energy consumption—ultimately limiting deployment in latency-critical or power-constrained scenarios. In contrast, ISMC architectures collapse sensing, storage, and processing into a unified physical substrate, enabling dramatic improvements in energy efficiency, throughput, system latency, and integration density. A comprehensive overview of these core advantages is summarized in Fig. 5.

#### Energy Efficiency

A primary advantage of ISMC lies in its ability to reduce energy consumption by eliminating redundant data movement. Because data are processed where they are generated, energy efficiency—typically quantified in joules per bit (J/bit)—can approach biological levels. Memristor-based ISMC devices have demonstrated sub-10 fJ/bit operation, comparable to synaptic energy expenditure (Table 1). Similarly, the nanowire-based optoelectronic synaptic transistors achieve energy efficiencies up to 0.7 fJ [44]. All-optical graphene/WSe_2_ synaptic devices can further minimize this energy expenditure to ~ 127 aJ purely through light-intensity switching [45]. These advances are particularly impactful for ultra-low-power domains such as distributed IoT nodes, wearable electronics, and autonomous sensor networks, where energy efficiency directly determines system lifetime and scalability.

**Table 1:** Comparison of Performance Metrics Between Emerging and Conventional Memory Devices. Reproduced from Ref. [57] with permission from Nature, Copyright 2025

	Emerging Memory Devices				Conventional Memory Devices		
	RRAM	STT-MRAM	FeFET	PCRAM	SRAM	DRAM	Flash
Area (F^2^)	< 4(3D)-12	6–20	4–16	4–20	> 100	6	4–10
Read/Write Voltage (V)	< 3	< 1.5	4	< 3	< 1	< 1	> 10
Power Consumption (pJ/bit)	~ 0.1	~ 0.1	~ 0.01	~ 10	~ 0.001	~ 0.01	~ 0.01–100
Read Time (ns)	< 10	< 10	< 20	< 10	~ 1	~ 10	10^4^–50
Write Time (ns)	< 10	< 5	< 20	< 50	~ 1	~ 10	10^4^–10^7^
Endurance	> 10^6^–10^12^	> 10^15^	> 10^5^	> 10^9^	> 10^16^	> 10^16^	> 10^4^
Non-Volatility	Yes	Yes	Yes	Yes	No	No	Yes
Scalability	Yes	Yes	Yes	Yes	Yes	Yes	Yes

#### Computing Speed

ISMC architectures dramatically accelerate data processing through in situ computation, bypassing multistage analog–digital conversion pipelines [46]. Optoelectronic memristors exemplify this advantage: They can sense photons, transiently store visual information, and perform weighted computation within the same device stack. By collapsing the conventional chain of “optical → electrical → digital” conversion, optoelectronic devices provide intrinsic parallelism and fast physical-domain operations. A processing speed of 11 TOPS (tera-operations per second) was achieved by using a microcomb-based photonic convolutional accelerator [47], while the recent diffractive architectures all-analog chip combining electronic and light computing (ACCEL) has demonstrated a system-level throughput of 4.6 Peta-OPS [48]. As a result, ISMC platforms can outperform traditional architectures in real-time applications such as high-speed vision, motion detection, and event-driven signal capture, where microsecond-level responsiveness is essential.

#### System Latency

System latency in von Neumann architectures originates not from computation itself but from data travel—through sensor front ends, memory hierarchies, caches, and buses—before reaching a processing unit. ISMC systems, by contrast, operate on a design principle of “processing at the source.” The 6 × 6 retinal-inspired ISMC array by Gong et al. [49] embodies this by performing recognition tasks concurrently with image acquisition, fully avoiding the costly transmission and conversion pipeline of traditional vision systems. Chen et al*.* [48] achieved a sensing-processing latency of just 72 ns using an all-analog optical computing architecture. The orders-of-magnitude acceleration of processing time over conventional CMOS pipelines enables real-time resolution of ultrafast motion. The ultra-low latency of ISMC system behavior is critical for autonomous driving [50], industrial process control [51], robotics [52], and remote healthcare [53], where delayed responses can directly compromise safety or system performance.

#### Integration Density, Scalability, and Security

Conventional architectures require physically distinct components—sensor arrays, memory modules, and processors—leading to large area overhead and challenging scaling. ISMC’s functionally unified design enables substantially higher integration density. The all-hardware mCNN reported in ref. [29] achieves a performance-per-area density 30 × higher than that of commercial GPUs, underscoring ISMC’s potential for compact, high-performance edge hardware. Beyond integration density, ISMC architectures inherently support scalability and security. By distributing computation across device arrays, performance scales nearly linearly with array size, enabling flexible deployment from miniature edge sensors to larger autonomous systems [54, 55]. Moreover, processing data near its point of origin minimizes on-chip transmission paths, reducing exposure to interception or tampering and offering natural advantages for secure computing applications such as financial analytics and classified information processing [42, 56].

Tables 1 and 2 summarize representative ISMC demonstrations across different material systems, device architectures, and sensing modalities. Reported metrics include energy consumption per operation, sensing-to-processing latency, array density, and task-level accuracy, providing a comparative overview of current capabilities. Emerging devices such as RRAM, spin transfer torque-magnetic random access memory (STT-MRAM), FeFETs, and PCRAM offer superior scalability, non-volatility, and low programming energy relative to static random access memory (SRAM), dynamic random access memory (DRAM), and flash—properties directly aligned with ISMC’s architectural requirements. Neuromorphic accelerators based on these devices further demonstrate favorable energy efficiency (TOPS W^−1^) and area efficiency (TOPS mm^−2^) across benchmark datasets. Notably, the performance variances among these ISMC chips stem from several architectural and physical factors. First, ISMC architectures demonstrate an intrinsic architectural advantage, where systems utilizing mature CMOS process nodes can still achieve energy and area efficiencies that substantially outperform traditional digital baselines on advanced nodes, effectively circumventing the conventional process wall. Second, the variations in task accuracy and computational efficiency reflect an inherent trade-off between device-level analog precision and system-level performance. Because ISMC systems directly execute computations in the analog domain, emerging NVMs inherently operate with lower or mixed bit precision compared to highly precise digital SRAMs. Fortunately, the deployment of algorithm-hardware co-design effectively preserves competitive inference accuracy. Finally, emerging NVMs possess distinct advantages in area efficiency, enabling higher effective storage capacities within constrained die areas.

**Table 2:** Performance Comparison of ISMC Chips. Reproduced from Ref. [58] with permission from Advanced Devices & Instrumentation, Copyright 2024

Chips	ReRAM	ReRAM	ReRAM	PCRAM	MRAM	SRAM
Neuron network	ResNet-20	CNN	CNN	ResNet-9/MLP	CNN	ResNet-50
CMOS Process node (nm)	22	55	130	14	22	16
Storage capacity (Mb)	4	1	0.155	-	0.128	5
Input/Weight/Output precision (bit)	1–2-4/4–4-10/8–8-14	1–3-4/2–3-4	1–3-1	8–4-8	1–1-4	8–8-8
Energy efficiency ratio (TOPS/W)	195.7/47.26/11.91	53.17/21.9	78.4	10.5	5.1	9.5
Area efficiency(TOPS/mm^2^)	32.6/7.88/1.99	7.09/2.92	3.59	1.59	2.27	1.29
Accuracy (dataset)	92.1% (CIFAR-10); 67.17% (CIFAR-100)	98.8% (MNIST); 88.52% (CIFAR-10)	94.4% (MNIST)	98.3% (MNIST); 85.6% (CIFAR-10)	90.1% (CIFAR-10)	100% (ImageNet)

Traditional indicators like core-level throughput (TOPS) or computational efficiency (TOPS W^−1^) often neglect the substantial energy and latency overheads associated with the sensor interface and ADC, which can account for over 80% of the total power consumption in conventional pipelines. Consequently, comparing ISMC solely against digital baselines using component-level metrics may underestimate its systemic value. While recent initiatives like the NeuroBench framework [59] have made significant strides in standardizing neuromorphic benchmarks, they often focus on algorithmic or general system levels. To bridge this gap, we advocate for an Application-Centric Benchmarking Framework grounded in three core principles.

1. Energy-to-Decision (EtD). Moving beyond device-level “Joules per bit,” this metric quantifies the total energy required to convert a physical stimulus into a valid inference result. By encompassing the entire signal chain, EtD explicitly captures the energy savings gained by eliminating redundant ADC and data shuttling [9]. The system energy is defined as:
  1 $$E_{{{\mathrm{sys}}}} = E_{{{\mathrm{sensing}}}} + E_{{{\mathrm{computing}}}}$$
  where ${E}_{sys}$ is the total energy consumption of the system for a single decision event. ${E}_{sensing}$ is the energy consumed by the sensor to capture the physical stimulus. ${E}_{computing}$ is the energy required for processing the data and generating the inference result.
2. Effective Bandwidth Compression (EBC). EBC serves as a key indicator of an ISMC system’s ability to alleviate the “bandwidth wall” in localized edge processing scenarios. It is defined as the ratio between the raw sensory data volume and the transmitted feature vector size:
  2 $${\mathrm{EBC}} = \frac{{{\mathrm{Data}}_{{{\mathrm{raw}}}} }}{{{\mathrm{Data}}_{t} }}$$
  where $Dat{a}_{raw}$ refers to the volume of the original raw sensory data collected by the front-end. $Dat{a}_{t}$ represents the size of the feature vector or decision result that is actually transmitted off-chip or to the next stage.
3. Task-Level Accuracy vs. Efficiency Trade-off. Given the intrinsic stochasticity of analog substrates, benchmarks need to map the Pareto frontier between task precision and resource consumption. This approach allows for a fair comparison, highlighting scenarios where approximate computing offers superior utility over high-precision digital baselines [60].

In summary, the holistic advantages of ISMC encompass system-level EtD efficiency, low latency, and EBC. These capabilities position it as a central technological pathway for overcoming the long-standing bottlenecks of post-Moore computing. As breakthroughs in memristive materials, 3D heterogeneous integration, and algorithm-hardware co-design continue to converge, the establishment of such standardized benchmarking protocols will be defining for the scalable deployment of next-generation intelligent systems.

## Global Development Landscape of ISMC

The global landscape of ISMC technology has entered a phase of accelerated expansion, driven not only by architectural efficiency gains relative to conventional von Neumann models but also by escalating societal demands for high-performance, energy-frugal computation at the edge. ISMC is no longer positioned merely as a device-level curiosity; it has rapidly evolved into a strategic frontier where nations are racing to secure technological sovereignty in the post-Moore era.

**Fig. 6:**
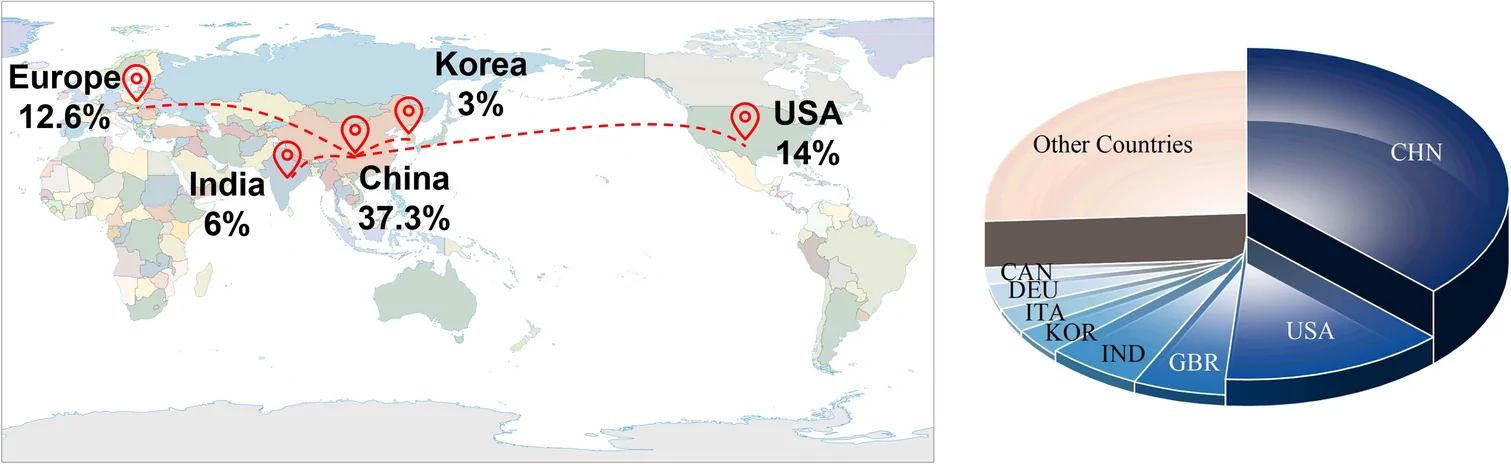
Publication distribution from 2015 to 2025. Data were retrieved from the Web of Science Core Collection using the search keywords including “in-sensor-memory computing,” “in-sensor computing,” or “neuromorphic computing,” with the publication date set from January 1, 2015, to December 3, 2025. The base world map is from China Standard Map Service (http://bzdt.ch.mnr.gov.cn/. No. GS(2016)1663)

A survey of all 3,681 ISMC publications from the Web of Science Core Collection from January 1, 2015, to December 3, 2025, reveals that synaptic materials, multimodal neuromorphic sensing, and heterogeneous integrated chip architectures represent current hotspots [61]. Globally, the primary regions driving ISMC development are East Asia, North America, Europe, and India (Fig. 6). The semiconductor manufacturing infrastructure provides a strong industrial backbone, enabling fast iteration of FeFETs, domain-wall memory, and 3D-integrated neuromorphic logic tailored for commercial integration. Beyond these centers, Malaysia [62], Russia [63], Switzerland [60], and Saudi Arabia [64] have also entered the field, with research agendas aligned to local strengths such as flexible electronics, oxide semiconductors, and specialized sensing modalities. Collectively, these efforts demonstrate that ISMC has transitioned from academic exploration to a multi-region, multi-industry competition for leadership in materials, architectures, algorithms, and system deployment.

Meanwhile, the maturation of ISMC technology increasingly hinges on the vertical coordination of the entire innovation chain—from neuromorphic materials and device physics to software architectures and multimodal fusion algorithms. Technological breakthroughs are no longer isolated events; instead, they accumulate incrementally and synergistically, forming a hierarchical evolution pattern: materials → devices → 3D architectures → intelligent algorithms → full-stack systems → domain-specific applications. This layered progress is reshaping how sensing, memory, and computation interplay at the chip and system levels, gradually pushing ISMC from theoretical constructs toward scalable industrial deployment.

### Architecture Innovation for ISMC Systems

**Fig. 7:**
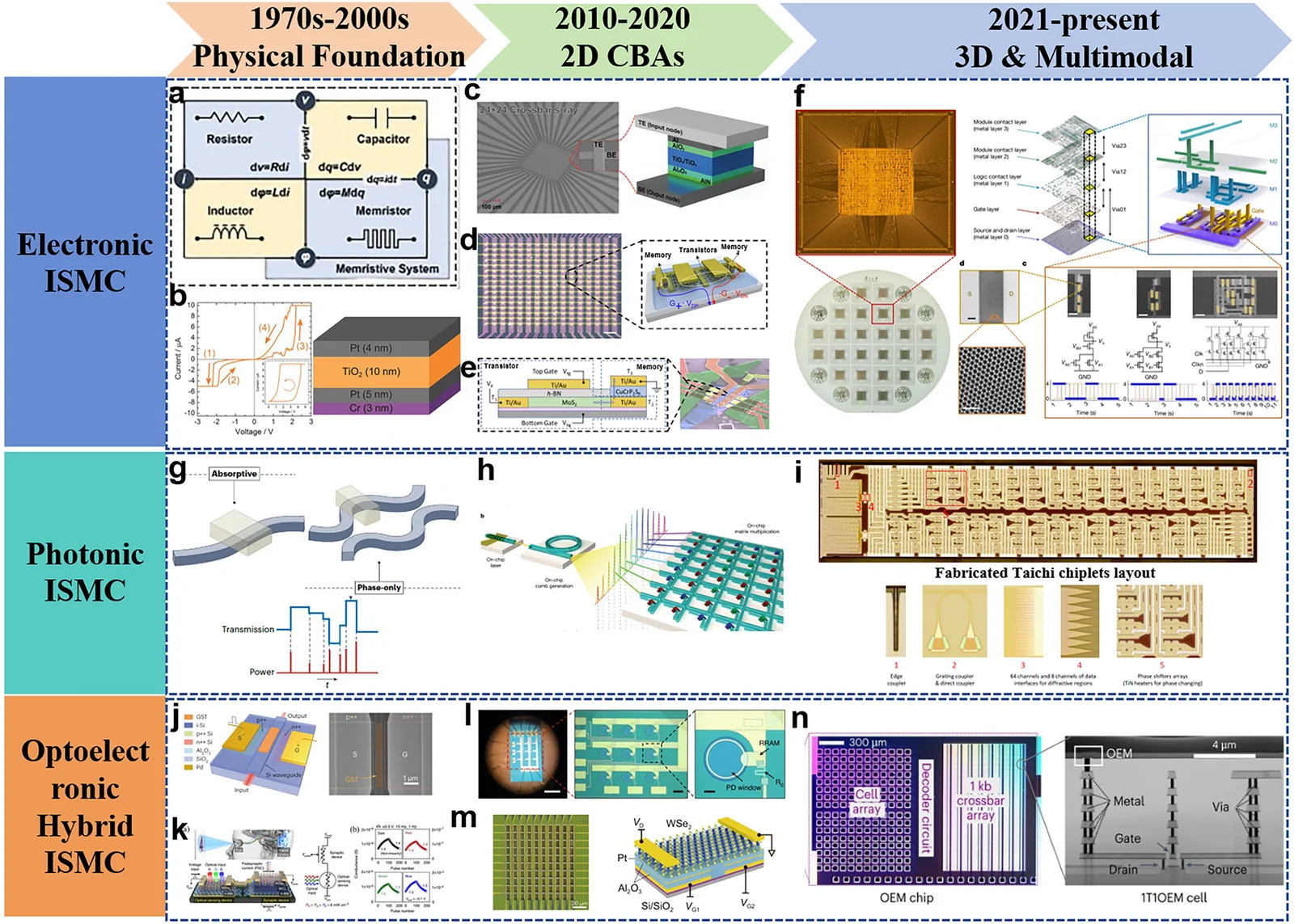
Typical architecture of electronic, photonic, and optoelectronic hybrid ISMC systems. **a** Theoretical foundation and **b** metal–insulator–metal (MIM) structure of electronic memristive devices. Reproduced from Ref. [71] with permission from Advanced Functional Materials, Copyright 2015. 2D CBAs of **c** RRAM. Reproduced from Ref. [72] with permission from Advanced Functional Materials, Copyright 2023, **d** nTnR memristor. Reproduced from Ref. [73] with permission from Nature Communications, Copyright 2025, **e** 2D vdW-heterogeneous 1T1R memristor. Reproduced from Ref. [74] with permission from Nano Letters, Copyright 2025. **f** A RISC-V 32-bit microprocessor paving the way for monolithic 3D integration—RV32-WUJI. Reproduced from Ref. [75] with permission from Nature, Copyright 2025. **g** Optical memristive platforms for non-volatile transmission modulation. Reproduced from Ref. [68] with permission from Nature Photonics, Copyright 2023. **h** Conceptual illustration of a fully integrated photonic architecture to compute convolutional operations. Reproduced from Ref. [69] with permission from Nature, Copyright 2021. **i** Taichi. Reproduced from Ref. [70] with permission from Science, Copyright 2024. **j** Electrical control and optical readout mode. Reproduced from Ref. [68] with permission from Nature Photonics, Copyright 2023.** k** Electro-optical co-modulated mode. Reproduced from Ref. [76] with permission from Nature Communications, Copyright 2024. **l** Integrated photodiodes with RRAM units. **m** 2D CBAs with split floating-gate 2D WSe_2_. Reproduced from Ref. [25] with permission from Light: Science & Applications, Copyright 2025. **n** ISMC chip based on 1 kb 1 T-1OEM array with Si CMOS circuits. Reproduced from Ref. [8] with permission from Nature Nanotechnology, Copyright 2024

The emergence of IMC provided the architectural seed for ISMC systems by collapsing the rigid separation between memory and computation that has constrained the von Neumann paradigm for over half a century. The discovery and later fabrication of the first memristive nanodevice by HP Labs in 2008 [65] converted Leon Chua’s 1971 theoretical construct [66] into a plausible hardware for massively parallel, analog-domain computation (Fig. 7a, b). Advancements in materials and fabrication processes led to the development of the 1T1R architecture—where a CMOS transistor is connected in series with a RRAM cell. This innovation realized the 2D CBAs, addressing the cross-talk issues plaguing pure RRAM arrays. Mainstream 2D CBA architectures now include RRAM CBA, n-transistor n-RRAM (nTnR) CBA, and memtransistor CBA (Fig. 7c, d). The advent of the memtransistor architecture introduced multi-terminal control capabilities, supporting complex synaptic plasticity while suppressing crosstalk without the need for additional selector devices [67]. Meanwhile, 1T1R array scales expanded to 32 × 32 and 64 × 64 configurations. In recent years, 3D vertical integration has transcended planar limitations and has propelled ISMC architectures from “laboratory validation” to “scenario-based deployment.” By vertically stacking multiple 2D arrays with metal via interconnects, 3D CBAs achieve an integration density 3–6 times higher than their 2D counterparts (Fig. 7f). Furthermore, their operational mechanism closely mimics the cerebral cortex’s layered and columnar structure, enabling high-density interconnections and efficient information processing.

The photonic ISMC architecture accomplishes the acquisition, feature extraction, and computational processing of sensing signals entirely within the optical domain, requiring no electro-optical conversion throughout or only a single conversion at the terminal. This design fully leverages the inherent advantages of photons, including high bandwidth, low latency, and parallel transmission [68]. Phase-change material (PCM)-based optical memristors represent the only mainstream technological platform capable of fully optical modulation and operation to date. Compared to electrically controlled PCM devices, all-optically controlled PCM memristors offer lower switching energy consumption, higher cycling endurance, and greater storage bit capacity. Furthermore, optical energy can be transferred to PCMs more efficiently, eliminating the thermal fatigue issues associated with electrical control. Feldmann et al. [69] demonstrated that the integrated photonic tensor core, which is constructed with PCM memory arrays and photonic chip-based optical frequency combs, can execute parallel convolution processing at a speed and energy efficiency far exceeding that of electronic GPUs (Fig. 7h). To support artificial general intelligence (AGI), a large-scale photonic chipset, Taichi, which is based on integrated diffraction-interference hybrid design and a universal distributed computing architecture [70] (Fig. 7i), was proposed. The chip boasts millions of neuron-equivalent capabilities and achieves 160 TOPS W^−1^. Additionally, it achieved an on-chip test accuracy of 91.89% on the 1623-category Omniglot dataset and generated high-fidelity AI content with efficiency improvements of up to two orders of magnitude. It paves the way for large-scale photonic computing and advanced tasks.

The optoelectronic hybrid ISMC architecture ingeniously combines the advantages of photonics and electronics, encompassing two primary modes: 1) Electrical control and optical readout mode. Based on the ferroelectric, MEMS, magneto-optical, or charge-trapping systems, the optical properties of materials are modulated by electrical signals, while optical signals are solely used for readout or transmission. This represents the mainstream form of optical memristors today, offering the key advantage of seamless integration with CMOS electronic systems. It is well suited for scenarios such as all-optical domain computing, high-speed optical communication, optical memory, and dynamic fine-tuning of photonic integrated circuits (PICs). Pan et al. [25] successfully integrated photodiodes with RRAM units, constructing a reconfigurable optoelectronic output unit capable of directly performing intelligent tasks with low power consumption and latency (Fig. 7l). However, the optical ISMC architecture currently faces two core challenges: the lack of efficient, low-loss, and scalable non-volatile photonic memory and the difficulty in implementing efficient nonlinear activation functions within the optical domain, which typically still relies on electro-optical conversion. 2) Electro-optical co-modulated mode. Synaptic potentiation is only triggered when both electrical and optical input signals are present, neither signal alone can alter the memristive state. This mode is adaptable to scenarios such as complex neuromorphic computing, nonlinear photonic computing, and optoelectronic hybrid ISMC. Peng et al. [76] proposed a hardware platform replicating the human visual pathway, comprising CBAs with split floating-gate 2D WSe_2_ unit devices and associated peripheral circuits that replicate the connectomics between the retina and visual cortex (Fig. 7m). Huang et al. [8] developed a multimodal optoelectronic memristor array for ISMC, establishing a monolithically integrated ISC prototype system for processing multistage visual tasks (Fig. 7n). This system integrates a 1 kb (1024 units) one transistor-one optoelectronic memristor (1T-1OEM) array with Si CMOS circuits on a single chip. The novel TiO_x_/ZnO-based OEMs offer multiple operating modes, e.g., electronic memristor (EM), dynamic optoelectronic memristor (D-OEM), and non-volatile optoelectronic memristor (NV-OEM), which can be effectively modulated through changes in charge density distribution induced by optoelectronic excitation.

Clearly, the essence of ISMC architecture innovation lies in the blurring of functional boundaries and the upgrading of integration dimensions. It evolves from the “separation of sensing, memory, and computation” to “hardware-level deep fusion,” and from “2D planar integration” to “3D vertical expansion.” Each breakthrough relies on the synergistic advancement of materials, devices, and arrays and targets to construct neuromorphic hardware characterized by high density, high energy efficiency, multimodality, and fault tolerance. It provides essential support for breaking through the von Neumann bottleneck and realizing AGI.

### Material and Device Foundations of ISMC

Materials are critical to device performance, as their dimensions and functional types determine the structure and performance of ISMCs. Here, we have summarized the development of materials and their corresponding functionalities, along with the corresponding device types.

**Fig. 8:**
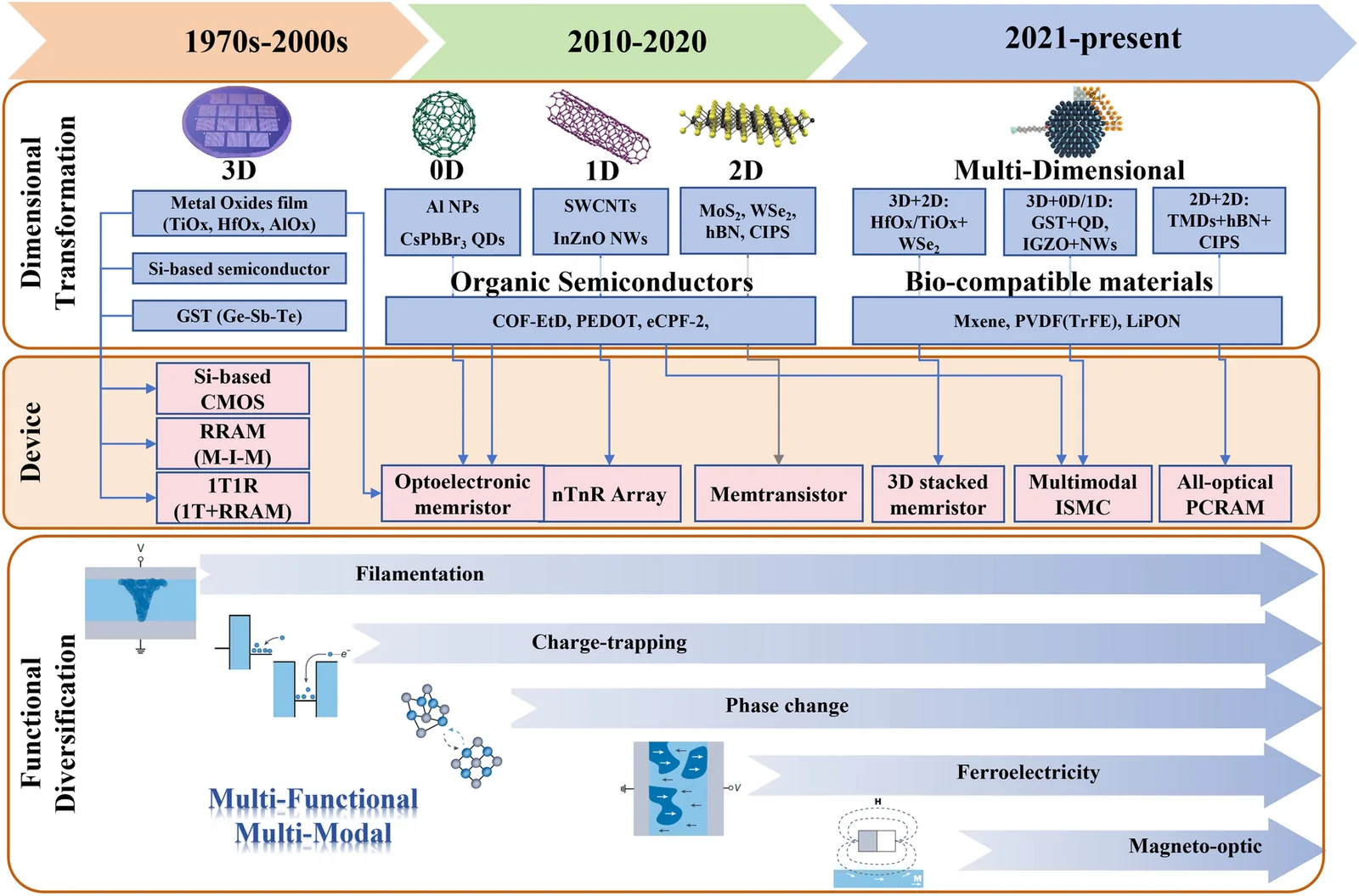
Evolutionary roadmap of materials and devices for ISMC. 3D material, Reproduced from Ref. [84] with permission from Nature Electronics, Copyright 2020. 0D & 1D material, Reproduced from Ref. [85] with permission from Nature Materials, Copyright 2007. 2D material, Reproduced from Ref. [86] with permission from Nature Nanotechnology, Copyright 2011. Functional Diversification images Reproduced from Ref. [68] with permission from Nature Photonics, Copyright 2023

Along the axis of dimensional transformation in Fig. 8, ISMC materials have evolved from bulk Si-based platforms toward low-dimensional and heterostructured systems. Zero-dimensional (0D) materials (e.g., nanoparticles (NPs), quantum dots (QDs)), which are confined to the nanoscale across all three dimensions, exhibit prominent quantum confinement effects, can serve as charge-trapping centers, and are utilized for fabricating light-controlled, programmable synaptic devices; one-dimensional (1D) materials (e.g., carbon nanotubes (CNTs), nanowires (NWs)), constrained in two dimensions with one dimension extended, possess directional carrier transport properties and are suitable for use as low-resistance conductive channels; and 2D materials (e.g., graphene, MoS_2_), featuring atomically thin layers dominated by surface effects with band structures determined by layer number, can function as channels, tunneling layers, and more, making them the most widely applied low-dimensional materials [75, 77, 78]. For instance, quasi-non-volatile capacitorless DRAMs based on edge-contact MoS_2_ achieve ultra-low leakage, enabling 5-bit precision storage and over 8,500 s data retention [79]; 3D materials (e.g., bulk/film metal oxides), with macroscale dimensions in all three axes, exhibit bulk-phase carrier transport behavior, benefit from mature fabrication processes, are suitable for large-scale integration, and serve as the mainstream resistive switching layers of memristors [80]. In addition, organic semiconductors and biocompatible materials are currently being used in the form of nanoscale structures for fabricating ISMC devices [81].

In parallel, functional diversification has driven a transition from purely electrical switching mechanisms to optoelectronic and multiphysics coupling [82]. It is based on their operational mechanisms: ion migration-based devices, which achieve resistive switching through electric field-driven ion migration that forms or ruptures conductive filaments; phase-change devices, which utilize Joule heating to induce reversible phase transitions between crystalline and amorphous states in chalcogenide compounds; ferroelectric devices, which modulate current relying on polarization reversal of ferroelectric materials and exhibit high switching speed and reliability; and magnetization reversal-based devices, which regulate the magnetization direction of ferromagnetic layers via spin torque, based on the tunnel magnetoresistance effect [83].

Fig. 8 illustrates the evolutionary trajectory of material systems for ISMC. The development of ISMC materials has undergone three phases: the foundation phase (pre-2000s), where the resistive switching characteristics of 3D metal oxides and Si-based materials supported the fundamental validation of single-function devices such as two-terminal RRAM and Si-based transistors; the integration phase (2010s-2020), where the quantum effects and atomically sharp interface properties of 0D/1D/2D low-dimensional materials drove the evolution of devices from “single memory” to multifunctional devices integrating “light sensing, memory, and computing”; and the hybrid integration phase (2021–2025), where the complementary advantages of multi-dimensional composite materials directly empowered the implementation of 3D-integrated and multimodal sensory devices, achieving “simultaneous improvement in performance and integration density.” Consequently, by leveraging the characteristics of material dimensionality, precisely controlling charge and ion transport through stacking, doping, interface engineering, and defect engineering, and synergizing the advantages of materials with different dimensions, neuromorphic devices with low power consumption, high linearity, and uniformity can be realized, which support the efficient operation of ISMC systems.

### From Neuromorphic Devices to Intrinsic Intelligence

**Fig. 9:**
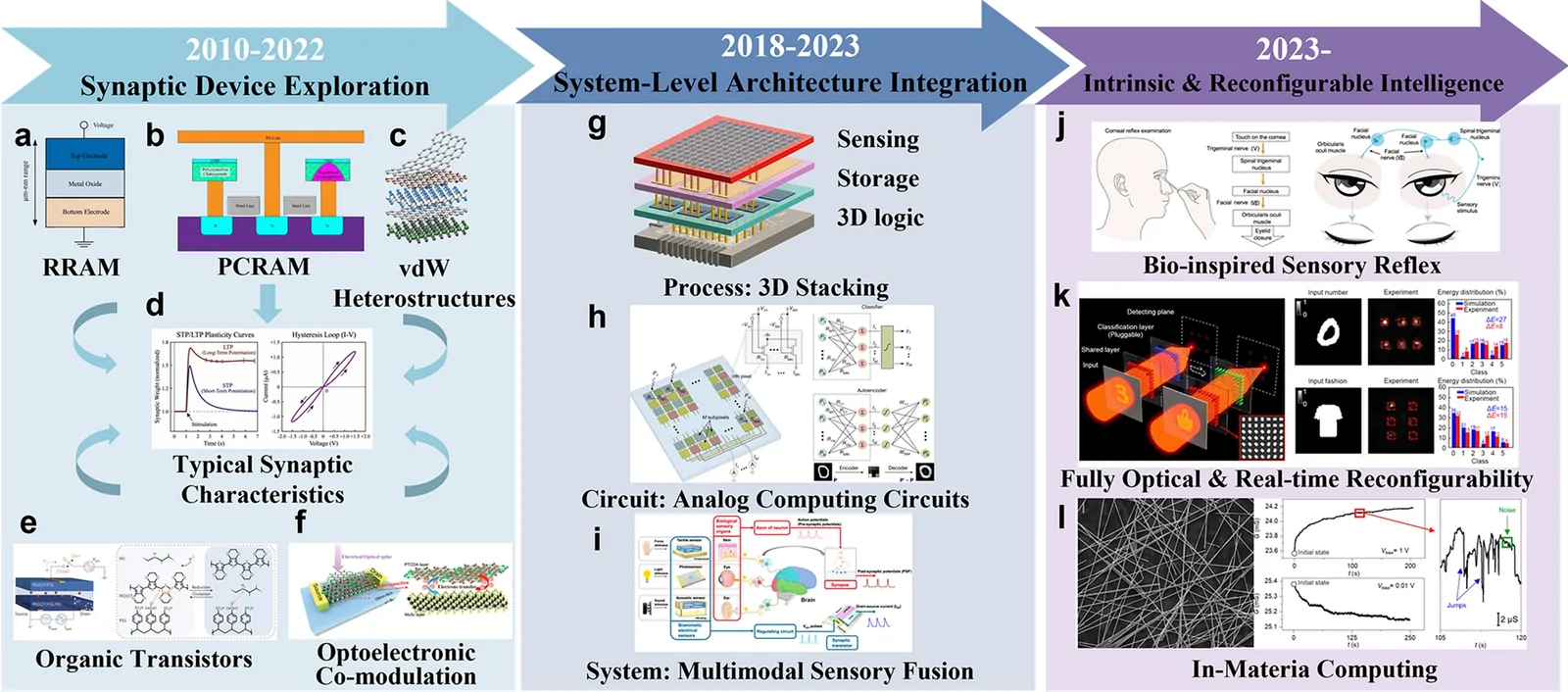
Chronological progression of neuromorphic innovation from synaptic devices to intrinsic intelligence. Schematic diagrams of **a** RRAM and** b** PCRAM. **c** vdW Heterostructures. Reproduced from Ref. [88] with permission from Nature, Copyright 2013. **d** Typical synaptic characteristics. **e** Organic Transistors, Reproduced from Ref. [89] with permission from Nature Materials, Copyright 2017. **f** Optoelectronic Co-modulation. Reproduced from Ref. [90] with permission from Advanced Materials, Copyright 2018. **g** 3D Stacking. Reproduced from Ref. [97] with permission from Advanced Functional Materials, Copyright 2026. **h** Analog Computing Circuits. Reproduced from Ref. [93] with permission from Nature, Copyright 2020. **i** Multimodal Sensory Fusion. Reproduced from Ref. [92] with permission from ACS Nano, Copyright 2021. **j** Bio-inspired Sensory Reflex. Reproduced from Ref. [95] with permission from Nature Communications, Copyright 2023. **k** Fully Optical and Real-time Reconfigurability. Reproduced from Ref. [96] with permission from Opto-Electronic Advances, Copyright 2024. **l** In-materia computing. Reproduced from Ref. [94] with permission from Nature Communications, Copyright 2025

The trajectory of neuromorphic device innovation, as delineated in Fig. 9, reflects a fundamental paradigm shift: moving from the emulation of basic biological functions to the realization of intrinsic, material-based intelligence [87]. This evolutionary progression can be categorized into three distinct yet overlapping phases.

The initial era 2010–2022 was defined by the quest for physical substrates capable of mimicking neural plasticity. During this period, research primarily focused on validating fundamental synaptic behaviors—such as long-term potentiation (LTP) and hysteresis—within emerging non-volatile memory technologies and low-dimensional materials [88]. While these efforts successfully demonstrated conductance tunability at the single-device level, they remained largely component-centric, serving as the building blocks for future systems [89, 90].

As device maturity improved, the field transitioned toward System-Level Architecture Integration (2018–2023). The emphasis shifted from optimizing individual components to constructing hierarchical systems through 3D stacking [91] and multimodal sensory fusion [92]. This architectural convergence bridged the gap between raw device physics and functional application, significantly reducing reliance on external processors and enabling efficient “compute-at-sensor” paradigms for edge scenarios [93].

Most recently, the domain has advanced into the phase of intrinsic and reconfigurable intelligence. Transcending fixed architectural designs, current innovations exploit the inherent nonlinearity and chaotic dynamics of materials to execute computation directly—a concept known as In-Materia Computing [94]. Coupled with bio-inspired sensory reflexes [95], these developments enable systems to process information with high-level autonomy [96]. This progression suggests that the next generation of ISMC devices will evolve from passive computational units into intelligent agents capable of adaptive, in situ learning.

### Neuromorphic Paradigms on ISMC Architectures

**Fig. 10:**
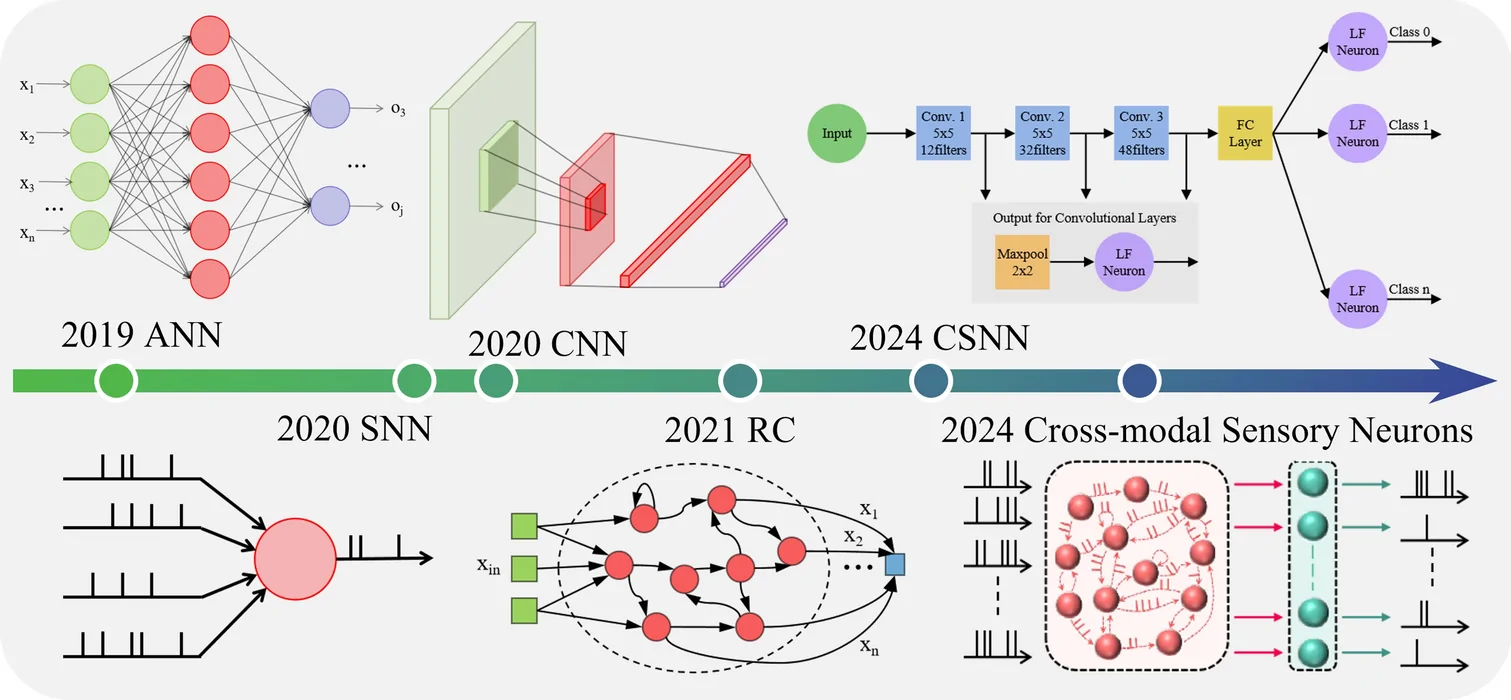
Evolution of neural networks from 2019 to 2025. Model of 2024 CSNN network architecture. Reproduced from Ref. [99] with permission from Nature Communications, Copyright 2024. The spiking reservoir network architecture of 2024 cross-modal sensory neurons. Reproduced from Ref. [102] with permission from Computer Science, Copyright 2024

A defining shift in the ISMC landscape is the growing recognition that designs of neuromorphic computing algorithms and corresponding neural network architectures are not peripheral to hardware innovation—they are structural determinants of whether ISMC hardware can transition from laboratory prototypes to scalable technologies. The progress in the field of neuromorphic computing, from CNN mapping to event-driven spiking neural networks (SNNs), and later to reservoir computing (RC) and cross-modal sensory neurons (Fig. 10), illustrates a deepening entanglement between device physics and computational abstractions. The integration of CNNs with memristor arrays accelerates matrix multiplication [29]. The emergence of SNNs enables spiking coding to achieve asynchronous event-driven operation in tandem with memristive neurons (e.g., Li_x_AlO_y_) [98]. RC breakthroughs leverage device dynamics as a physical reservoir to reduce training parameters [99]. Optimized co-design of lightweight artificial neural network hybrid models enables algorithm-hardware co-evolution (e.g., embedding convolutional kernels into memristor arrays [100]) and supports on-chip training. Xu et al. [101] proposed a CNN-SNN (CSNN) model, which integrates the feature learning capability of CNNs with the cognitive capacity of SNNs. This model learns the spatiotemporal representation of image encoding in an event-driven manner, significantly reducing both the number of required neurons and the volume of training samples. Recently, developments in cross-modal sensory neurons enable multi-sensory signal feature extraction within sensors, facilitating robotic environmental interaction [102].

Importantly, the co-evolution reflects an inversion of the traditional computing stack: Algorithms increasingly adapt to device physics, not the other way around. From 2020 to 2025, developments progressed from on-sensor preprocessing architectures to neuromorphic compilers and distributed learning frameworks. In 2022, Cui et al. [36] successfully implemented an on-sensor preprocessing architecture using ferroelectric photoconductive sensor arrays to achieve in situ image convolution, reducing backend data volume by 95%. In 2024, Yang et al. [103] developed the neuromorphic compiler “Tianmouxin” vision chip. The hybrid readout architecture achieved high-speed sensing at up to 10,000 frames per second and a dynamic range of 130 dB. To optimize hardware mapping, an energy-aware CSNN employs difference-of-Gaussian filters for polarity separation, allowing strictly positive weights to be directly mapped onto single 1T1R devices for area-efficient analog vector–matrix multiplication [104]. Instead of forcing emerging materials to mimic digital logic, researchers are now exploiting intrinsic material properties—temporal decay, hysteresis, wavelength selectivity—to construct new computational primitives. For instance, the intrinsic wavelength selectivity of graphdiyne/WSe_2_ heterostructures enables 100% accurate multi-color image classification and wavelength-adaptive reservoir computing [105]. This shift is enabling ISMC systems to execute neuromorphic computing tasks, e.g., in-sensor convolution, spatiotemporal coding, and on-chip learning, with unprecedented energy efficiency. The advancement of algorithms, in turn, helps unlock hardware potential. For instance, RC algorithms leverage device physics to reduce computational load [106], while co-design integrates CNNs into sensors to achieve zero-latency edge detection.

It is noteworthy that although neurocomputing and ISMC are highly intertwined in technology, they have distinct focuses in their core connotations. The essence of neurocomputing lies in simulating the basic principles of information processing in biological neural systems, emphasizing “how to compute like the brain.” While ISMC is a hardware integration paradigm, its core lies in integrating the three functions of sensing, memory, and computing at the physical level within the same device or architecture, with the emphasis being “where and in what form to implement the computation.” Therefore, ISMC provides a highly promising physical carrier for achieving neurocomputing. Its device has natural dynamic characteristics that are suitable for constructing pulse neurons and synapses, thereby efficiently running brain-inspired algorithms such as SNN. However, ISMC hardware can also run traditional CNNs and other non-pulse algorithms, and conversely, neurocomputing algorithms can also be implemented on non-ISMC cloud hardware. The current research trend is to achieve the deep integration of the principles of neurocomputing into the physical foundation of ISMC through the collaborative design of algorithms and hardware, thereby directly implementing high-efficiency spatiotemporal information processing at the sensing end. This evolution from “functional integration” to “principle fusion” indicates that the maturity of ISMC depends on the symbiosis of software and hardware, marking a profound transformation in this field from merely pursuing hardware miniaturization to building truly brain-inspired intelligent perception systems.

## Emerging Application Scenarios

The transition of ISMC from component-level research to system-level deployment addresses four fundamental constraints within the current Internet of Everything (IoE) landscape: strict latency requirements, bandwidth saturation, energy scarcity in remote environments, and data privacy. Distinct from centralized cloud computing models, ISMC architectures enable in situ processing at the sensory interface, thereby offering strategic advantages in latency-sensitive and resource-limited applications.

### Real-Time Autonomous Systems and High-Speed Machine Vision

In safety-critical autonomous systems such as high-speed drone navigation, robotic evasive maneuvers, and autonomous driving, the latency associated with conventional frame-based machine vision is a fundamental bottleneck [107]. ISMC architectures completely bypass this classical latency wall by executing continuous analog processing or event-driven computation directly at the sensory node. A breakthrough paradigm in this domain is the integration of diffractive optical and analog electronic computing. The recently developed ACCEL processes the optical field intrinsically as photons interact with the sensor, before any digital conversion occurs [48]. This architecture achieves an unprecedented sensing-to-processing latency of merely 72 ns and a system-level throughput of 4.6 Peta-OPS, making it exceptionally well suited for high-speed target tracking and real-time perception. Furthermore, retinomorphic ISMC devices offer a biologically plausible route to high-speed motion perception. Emulating the transient responses of biological retinal ganglion cells, these advanced sensors exclusively capture and process dynamic temporal variations (events), entirely ignoring redundant static backgrounds [108].

Recent demonstrations of computational event-driven vision sensors utilize in-sensor SNNs to directly convert dynamic motion into programmable, sparse spiking signals. By eliminating the digitization and transmission of redundant visual data, these hardware-level innovations achieve sub-microsecond responsiveness. Such ultra-low-latency capability is the fundamental enabler for next-generation agile robotics, allowing autonomous agents to perceive and react to complex, high-speed environments independent of external network reliability [109]. At the material and device level, the architecture employs WSe_2_ photodiodes configured with a floating split-gate structure, which allows for non-volatile, programmable photoresponsivity. A single pixel consists of two parallel photodiode branches with opposite polarities (PN and NP) and distinctly different photoresponse speeds. This hardware design intrinsically enables event-driven operation; the pixel outputs a zero net current under steady-state illumination but instantly generates transient spiking signals when local light-intensity changes. Algorithmically, these non-volatile and multilevel photoresponsivity states map directly to synaptic weights, facilitating the in situ execution of a SNN through physical matrix–vector product operations. At the system level, this tightly integrated pipeline yields profound benefits for autonomous perception: It reduces redundant static vision data generation by 98% compared to conventional frame-based sensors. Consequently, it achieves an ultra-low temporal latency of 5 μs and a 92% accuracy for dynamic motion recognition tasks, completely eliminating the latency and power consumption associated with transferring raw visual data to external processing units.

### Alleviating the Data Deluge in Smart Infrastructure

**Fig. 11:**
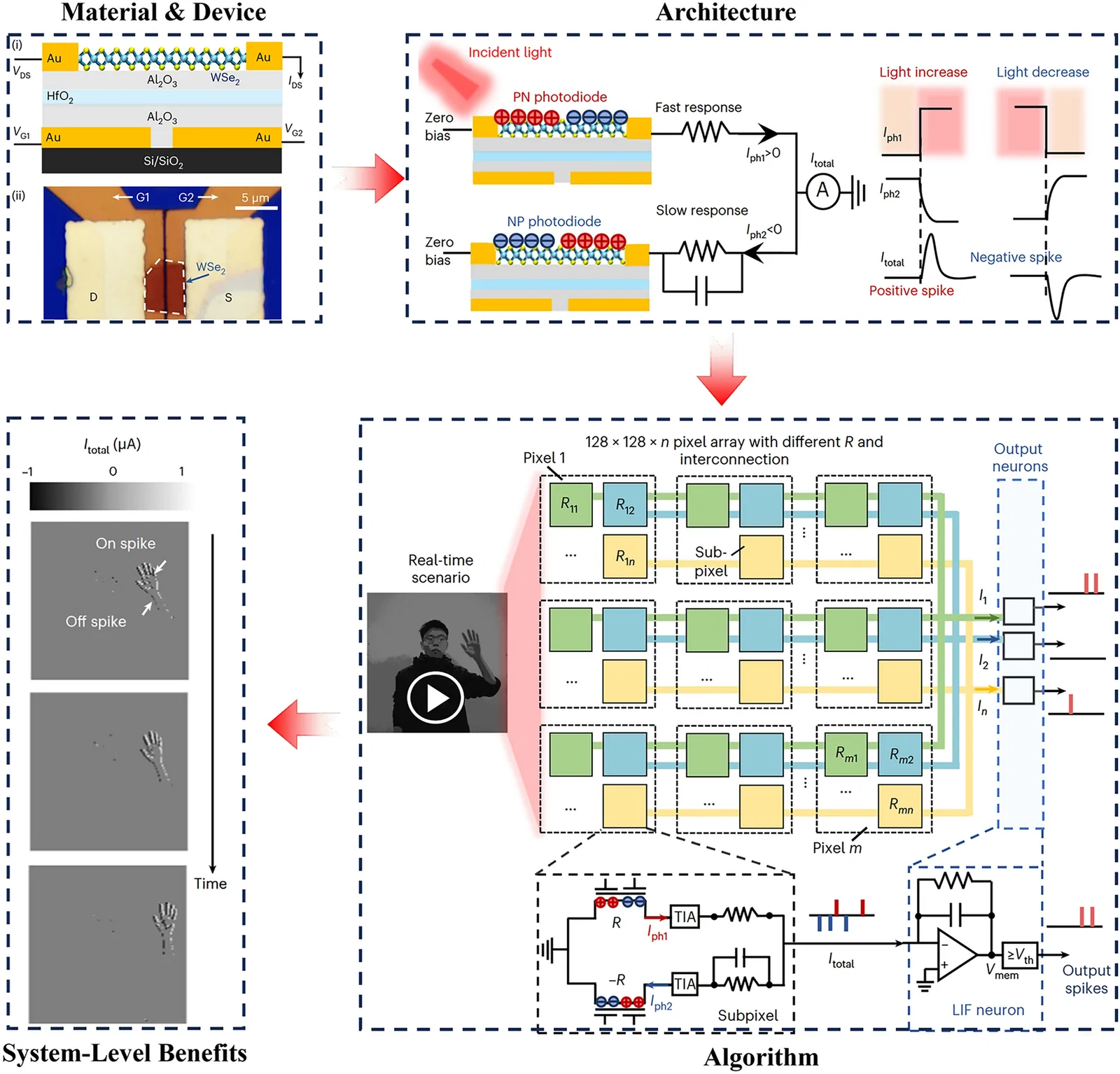
Translational pathway of an in-sensor SNN based on 2D material devices. Reproduced from Ref. [109] with permission from Science China Information Sciences, Copyright 2024

In data-intensive application scenarios, the continuous transmission of high-dimensional sensory data constitutes a formidable communication bandwidth bottleneck [107]. ISMC architectures fundamentally alleviate data deluge by functioning as physical information filters directly at the sensory interface. By employing in-sensor compressive sensing or feature extraction paradigms, these architectures transmit only highly semantic feature vectors or anomaly classifications to the backend, rather than massive and redundant raw pixel arrays (Fig. 11).

In the context of high-throughput industrial inspection, recent ISMC devices based on ferroelectric photodiodes have successfully demonstrated in-sensor data compression and reconstruction-free recognition. These devices maintain exceptional target classification accuracy at drastically reduced sub-sampling rates, successfully preserving over 96% accuracy at a mere 15.6% sampling rate during the identification of microscopic surface defects in high-speed steel manufacturing [110]. This reconstruction-free approach transmits only highly compressed feature representations rather than raw defect images, significantly relieving the bandwidth pressure on industrial IoT networks. Furthermore, in the aerospace remote sensing domain, Low Earth Orbit satellites face severely restrictive downlink bandwidth walls when transmitting massive hyperspectral images to ground stations. Recent breakthroughs in electrically tunable photodetectors and “spectral kernel machines” have enabled machine learning analysis of scene spectra directly in the analog domain [111]. By executing broadband in-sensor processing prior to ADC for the in situ identification of specific surface material signatures or chemical compositions, this novel computational paradigm completely circumvents the hyperspectral downlink bottleneck. Consequently, it establishes a highly bandwidth-efficient, hardware-level edge AI framework tailored for Earth observation and deep space exploration [112].

### Ultra-Low-Power Always-On Edge IoT and Extreme Environments

In geographically dispersed or extreme environments, such as deep space exploration, nuclear facilities, and remote industrial pipelines, edge IoT nodes are strictly constrained by energy scarcity and harsh physical conditions. Conventional “always-on” sensors continuously digitize and process background noise to identify rare anomalies, incurring unacceptable static power consumption. Furthermore, in true extreme environments characterized by intense radiation and ultra-high temperatures, traditional von Neumann CMOS architectures suffer from severe performance degradation and require costly, power-hungry radiation-hardening redundancy.

**Fig. 12:**
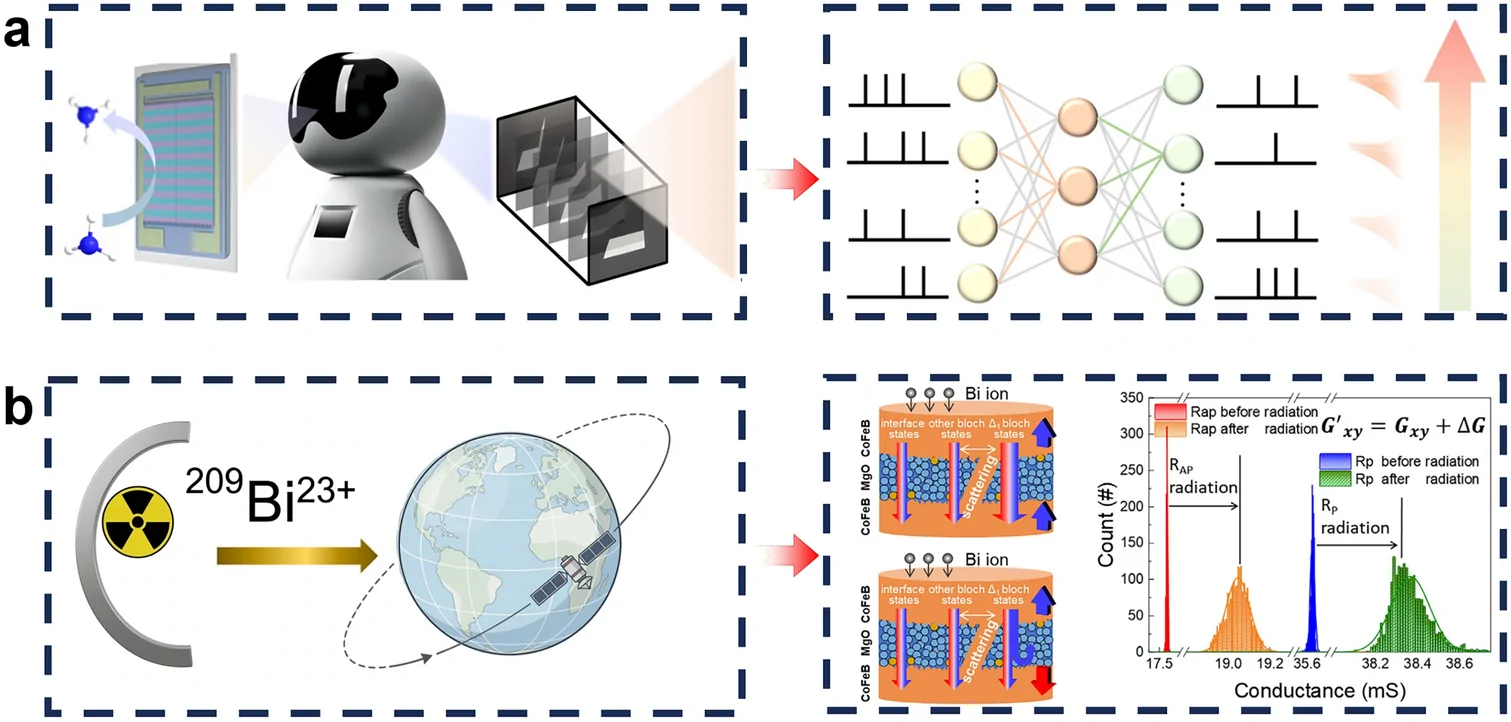
**a** Schematic diagram of SNN-resolved gas flow. Reproduced from Ref. [113] with permission from Research, Copyright 2026. **b** Application of spintronic neuromorphic hardware in extreme aerospace environments. Reproduced from Ref. [116] with permission from Applied Physics Letters, Copyright 2026

ISMC architectures circumvent these fundamental energy bottlenecks by executing analog feature extraction and event-driven classification directly at the sensing interface. The integration of advanced functional materials with neuromorphic arrays represents the paradigm for maintenance-free environmental intelligence. Recent breakthroughs have showcased ultra-low-power ISC platforms capable of functioning autonomously in distributed IoT networks. A prominent example is the reconfigurable neuromorphic olfactory memristor utilizing mixed-dimensional heterostructures, which can intrinsically compute and classify hazardous gas leakages using SNN, operating at near-zero standby power [113] (Fig. 12a). By functioning as energy-autonomous, event-driven smart nodes, these ISMC platforms facilitate the scalable and sustainable deployment of intelligent IoT surveillance in previously inaccessible locations.

Crucially, beyond terrestrial energy constraints, emerging ISMC technologies have demonstrated unprecedented resilience in radiation-intensive and high-temperature extreme environments. Recent research highlights that ISMC devices based on wide-bandgap materials, such as silicon carbide [114] and gallium oxide [115], can maintain stable neuromorphic reliability under extreme conditions, including temperatures exceeding 300 °C, strong radiation, and corrosive atmospheres. Moreover, breakthrough spintronic neuromorphic hardware, such as spin-orbit torque-magnetic tunnel junctions, not only exhibits intrinsic radiation hardness but can actively harness radiation-induced conductance fluctuations, as illustrated in Fig. 12b, to enhance neural network optimization for aerospace computing [116]. Ultimately, the intrinsic radiation resilience and energy autonomy of these ISMC architectures pave the way for robust intelligent perception in the most demanding frontiers.

### Native Privacy in Bio-Integrated Healthcare

In conventional wearable and implantable healthcare systems, the continuous transmission of high-dimensional physiological or neural data to centralized clouds constitutes a profound vulnerability. By executing machine learning inference directly within the sensory node, ISMC completely obviates the need for raw data transmission, transmitting only highly abstracted diagnostic outcomes, such as an arrhythmia alert or a decoded motor command, thereby establishing a robust hardware-level security boundary. For instance, a fully wearable ISC platform utilizing intrinsically stretchable organic electrochemical transistor arrays can perform real-time, analog-domain classification of complex biosignals while sustaining over 50% mechanical strain [6]. Flexible digital compute-in-memory chips have been developed using low-temperature polycrystalline silicon technology, integrating robust digital logic and memory arrays directly onto bendable wearables. This chip enables highly accurate (99.2%) local arrhythmia detection from ECG signals [117]. By executing edge computing directly at the skin interface, these systems drastically reduce reliance on external servers, ensuring that sensitive physiological data never leave the user’s physical perimeter.

### Multi-dimensional and Multimodal Perception

Unlike conventional vision sensors that primarily capture spatial light intensity, ISMC architectures natively integrate and process multi-dimensional optical parameters and multimodal physical quantities directly at the sensory terminal. [118, 119]. This hardware-level fusion eliminates the massive data overhead and complex software-level alignment required by traditional von Neumann architectures [120], enabling real-time, context-aware intelligence.

**Fig. 13:**
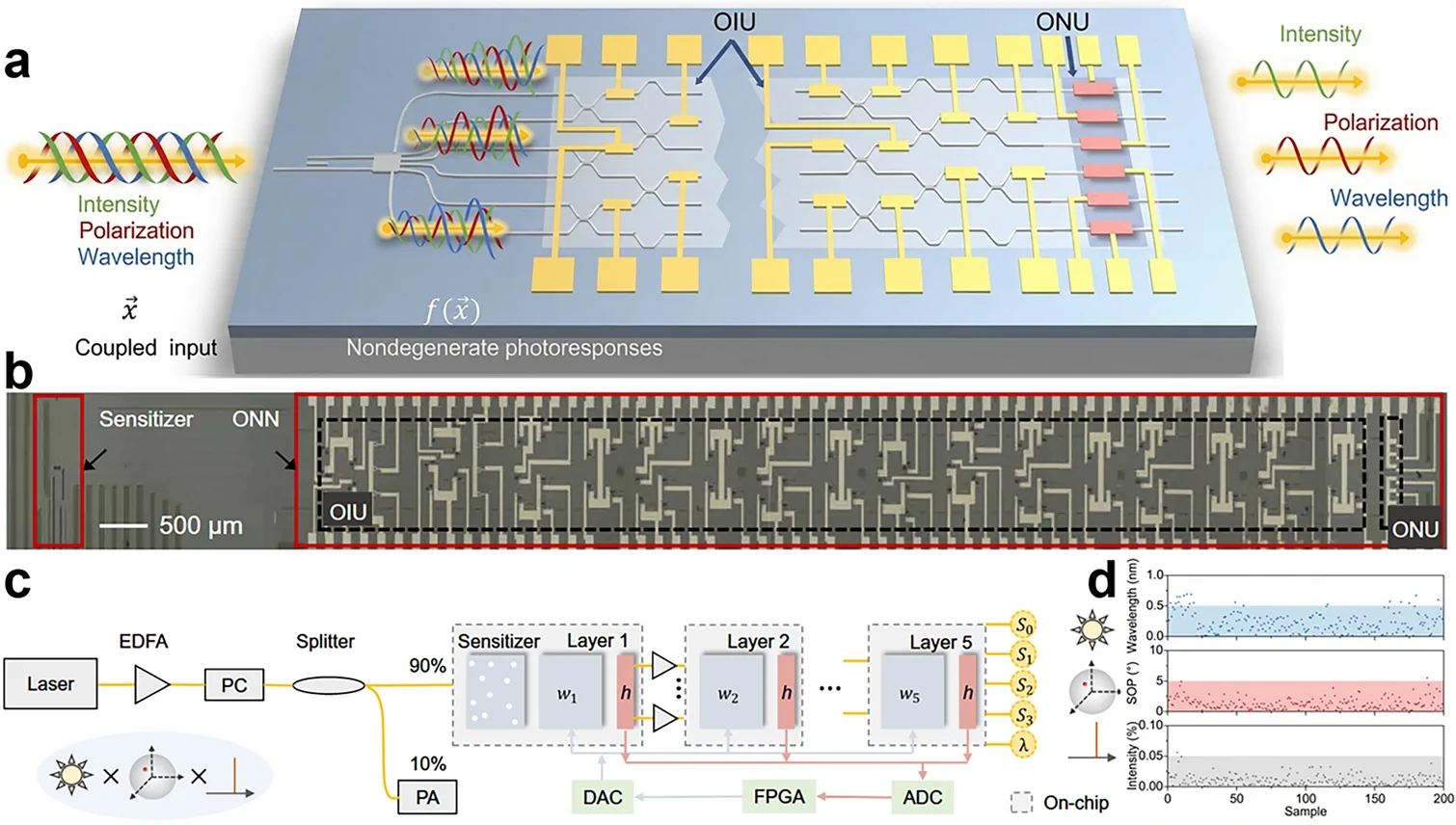
All-integrated multi-dimensional optical sensing based on a photonic neuromorphic processor. **a** Schematic architecture of the all-integrated chip. **b** Optical micrograph of the fabricated sensing circuit. **c** Experimental setup for the multi-dimensional optical sensing and in situ ONN training. **d** Decoupled accuracy evaluation. Reproduced from Ref. [121] with permission from Science Advances, Copyright 2025

Recent advancements in integrated photonic neuromorphic processors have enabled ISMC systems to decouple mixed visual stimuli natively. All-integrated multi-dimensional optical sensors can simultaneously map intensity ${\boldsymbol{I}}$, wavelength ${\boldsymbol{\lambda}}$, and polarization ${\boldsymbol{P}}$ into multi-channel outputs without interference [121]. As illustrated in Fig. 13, this is physically realized by pairing an optical sensitizer with an on-chip optical neural network (ONN), which directly processes and untangles the mixed incident light to achieve high-accuracy, interference-free multi-dimensional extraction. This capability addresses critical sensing bottlenecks in real-world applications such as autonomous navigation [122] and industrial inspection [123], where polarization and multi-spectral data can reveal surface defects or detect corona discharge [124] that conventional RGB intensity sensors fail to capture. Furthermore, by incorporating time ${\boldsymbol{t}}$ as a fundamental dimension, ISMC natively supports event-driven spatiotemporal processing [7]. By computing only dynamic changes and ignoring redundant static backgrounds, these systems drastically compress data bandwidth, mimicking the efficient spatiotemporal reflexes of biological retinas for high-speed motion recognition and trajectory tracking.

The cross-modal capability of ISMC architectures is critical for embodied intelligence and robotic manipulation, which intrinsically rely on the real-time coordination of diverse sensory inputs. For complex tasks such as grasping, advanced ISMC platforms have successfully integrated optoelectronic synapses with piezoelectric nanogenerators to create bio-inspired tactile-visual systems [125]. Furthermore, recent advancements in multimodal electronic skins have demonstrated the sensory capability to capture multi-dimensional vector forces [126], alongside temperature and material roughness [127]. Crucially, by migrating these multimodal sensory inputs into memristor-based ISMC architectures, systems can compute tactile features locally. Memristor-based neuromorphic perception enables a robotic hand to process tactile slipping features in situ, autonomously adjusting grip strength in real time to prevent slippage [128]. Similarly, in cross-modal wearable healthcare, flexible ISMC devices, such as those based on MXene or oxide-heterojunctions, have been developed to concurrently process visual, auditory, and tactile electrophysiological signals [120]. By cross-verifying multimodal inputs at the hardware level, these devices effectively mitigate misjudgments common in single-modal cognition, such as in complex gesture decoding, while keeping sensitive biometric data localized and secure at the physical edge [6].

### Toward System-Level Autonomy: Continuous, Closed-Loop, and Distributed Intelligence

**Fig. 14:**
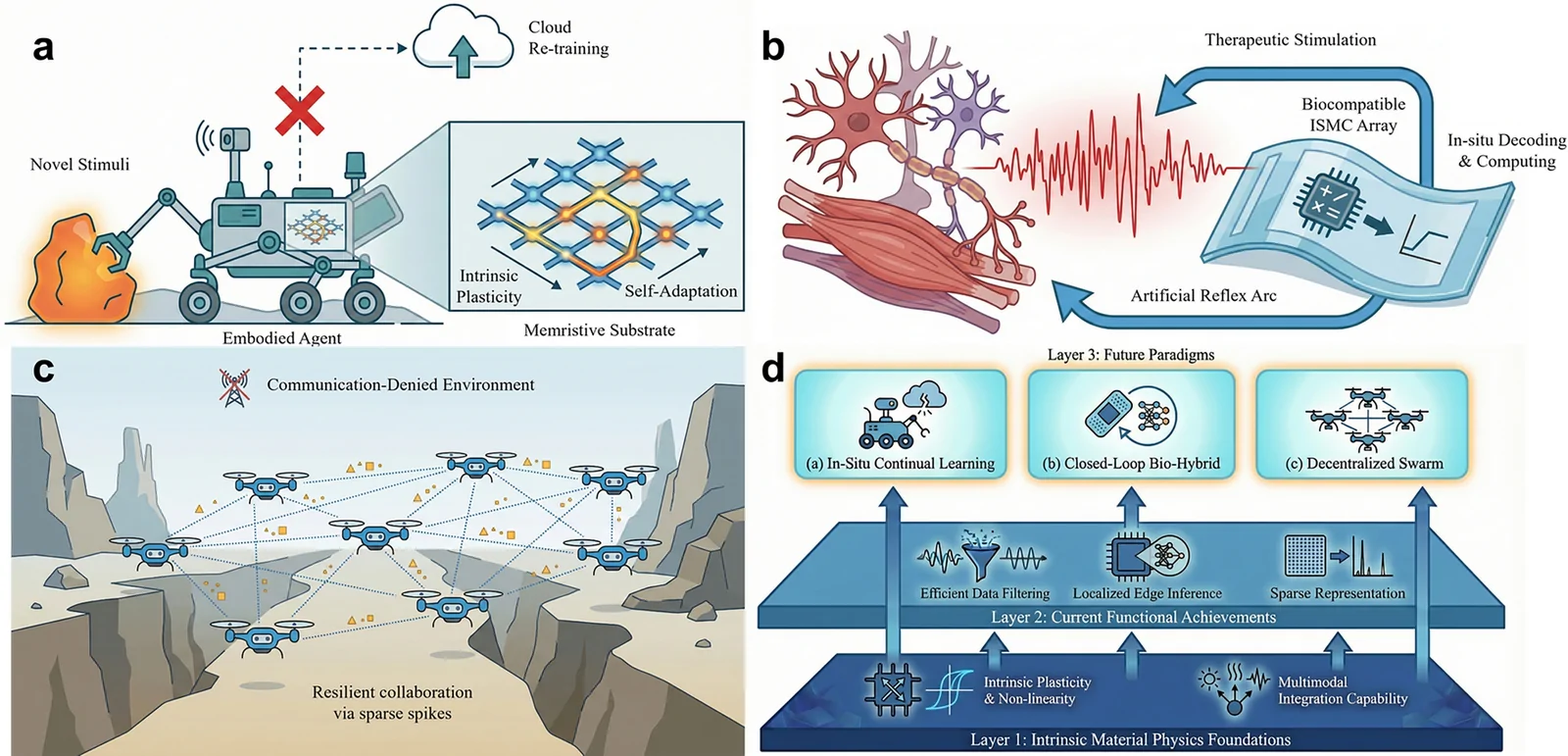
Future paradigms and enabling trajectories of ISMC toward system-level autonomy. **a** In Situ continual learning. **b** Closed-loop bio-hybrid interfaces. **c** Decentralized swarm perception. **d** Enabling dependencies

While Sects. 4.1–4.4 demonstrate ISMC’s current efficacy in localized inference and data filtering, its ultimate trajectory points toward dynamic, system-level autonomy. Grounded in ongoing advancements in intrinsic material intelligence and multimodal integration, we envision ISMC evolving from passive perception to active, system-level cognition. Figure 14 conceptually illustrates three transformative paradigms that represent the next frontier for ISMC deployment.

1. In situ continual learning. Current ISMC predominantly executes pre-trained algorithms at the edge. However, exploiting the intrinsic nonlinear dynamics and plasticity of memristive substrates will enable transition toward hardware-level continual learning. Rather than relying on cloud-based retraining, future embodied agents could autonomously adapt to environmental drifts or novel stimuli in real time, functioning as truly independent cognitive entities.
2. Closed-loop bio-hybrid interfaces. Future biocompatible ISMC arrays will not only decode complex electrophysiological signals but also compute triggering thresholds in situ to deliver immediate, localized therapeutic stimulation aimed at suppressing a tremor or seizure.
3. Decentralized swarm perception. As ISMC dramatically reduces bandwidth requirements by transmitting only sparse semantic spikes rather than raw data, it naturally unlocks the potential for massive multi-agent collaboration. In communication-denied or extreme environments, swarms of ISMC-equipped micro-robots could share abstracted environmental features over ultra-low-bandwidth channels, realizing resilient, decentralized swarm intelligence without centralized coordination.

## Industry–Academia–Research Ecosystem and Commercialization

The global deployment of ISMC technologies is now shaped by a unifying technological trajectory: materials-driven architectural reinvention, reinforced by rapid progress in multimodal integration, analog/neuromorphic signal pathways, and algorithm-hardware co-optimization. As ISC increasingly replaces conventional near-sensor architectures, ISMC is shifting from an exploratory research topic to a strategic platform technology underpinning next-generation intelligent systems. The acceleration of this transition reflects not only scientific advances but also a deepening alignment among industry, academia, and government-driven research programs worldwide.

**Fig. 15:**
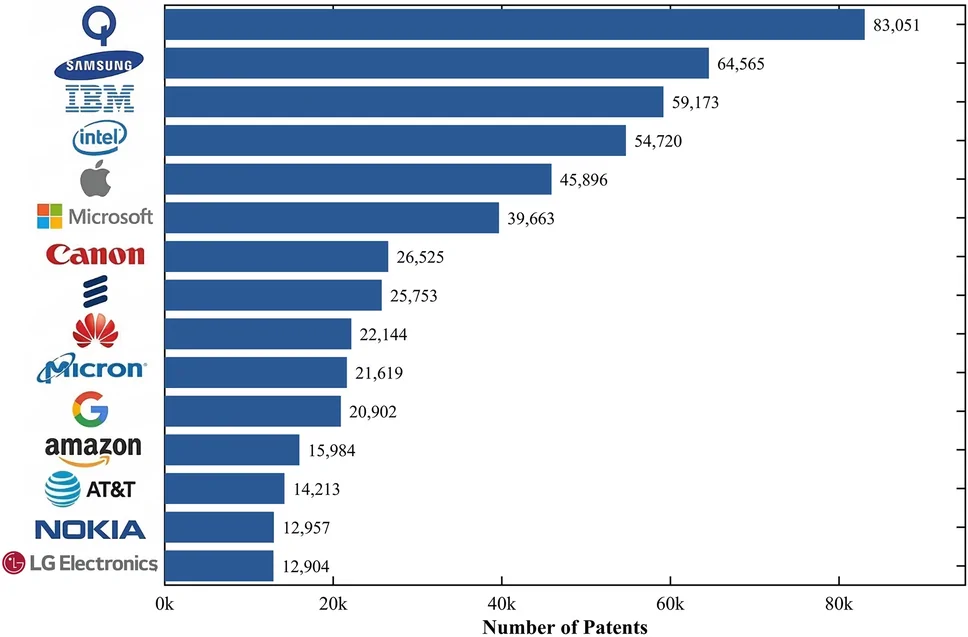
Top 15 global corporate patent holders in the field of ISMC and neuromorphic computing. The data were retrieved from the Lens.org database using the search keywords “In-sensor-memory computing,” “in-sensor computing,” or “neuromorphic computing,” covering the period from January 2010 to March 2026

An analysis of the global intellectual property (IP) landscape reveals aggressive patenting activities by major technology conglomerates. As shown in Fig. 15, a comprehensive patent search conducted via the Lens.org database identifies the top 15 global corporate patent holders. The quantitative distribution highlights a profound cross-industry convergence. Leading semiconductor and telecommunications enterprises, led by Qualcomm, IBM, Intel, and Ericsson, dominate the foundational hardware and communication IP, reflecting their strategic focus on establishing the underlying materials, devices, and architectures for post-von Neumann computing. Meanwhile, consumer electronics and Internet service leaders, such as Samsung, Apple, Microsoft, and Google, are rapidly accumulating patents. Their heavy investments signify a strategic push to secure edge AI, intelligent sensing algorithms, and system-level applications.

The USA has maintained a structural lead by deploying long-term programmatic funding mechanisms. Since 2008, agencies such as DARPA have systematically advanced the neuromorphic hardware ecosystem [129]—from the “Analog Computing-Based AI Processors” [130] initiative (2012) to the “Fast Event-based Neuromorphic Cameras and Electronics (FENCE)” [131] program (2020). These efforts illustrate a distinct US strategy: Cultivate foundational materials and device platforms first and then fuse them with emerging probabilistic, spiking, or event-driven computing paradigms to shape the next-generation intelligent hardware stack. Europe, by contrast, has pursued a materials-centric and coordination-driven model. Through the “Post-Moore Semiconductor Value Enhancement Strategy,” the European initiatives have mobilized 29 industrial partners across France, Italy, Germany, and the UK, catalyzing five major ISMC-related projects [132]. European research tends to emphasize physical modeling, device reliability, and materials physics, forming a deep scientific base that supports long-term industrial transition. Their ecosystem shows the classic European pattern: slower to market, but rigorous in fundamentals, particularly in ferroelectric, oxide, and hybrid perovskite device physics. East Asia—in particular China, South Korea, and increasingly Japan—has rapidly emerged as the most active innovation hub.

**Fig. 16:**
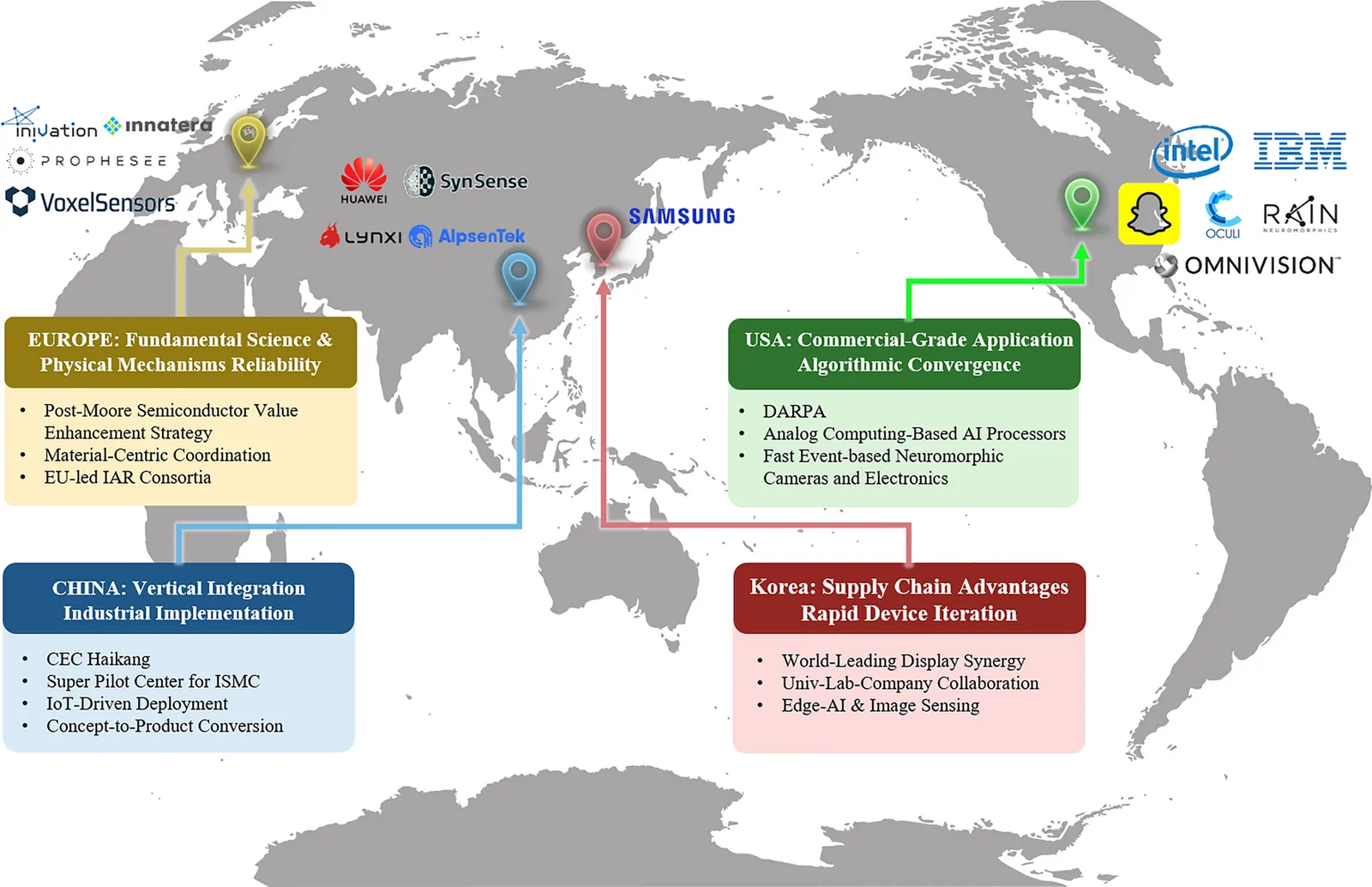
Global landscape of IAR strategies in ISMC. The base world map is from China Standard Map Service (http://bzdt.ch.mnr.gov.cn/. No. GS(2016)1561)

Global IAR collaboration has emerged as a decisive catalyst in the evolution of ISMC, fostering a diverse ecosystem where regional strategies complement each other, as shown in Fig. 16. In the USA, the ecosystem prioritizes commercial-grade applications and algorithmic convergence. Driven by initiatives from DARPA and leading technology enterprises (e.g., Intel, IBM), the region focuses on embedding ISMC functionalities into high-end AI accelerators and deploying analog computing-based processors, such as the Mythic AMP series, to achieve low-latency, event-driven computation. In Europe, the strategic focus is directed toward fundamental science and physical mechanism reliability. Through EU-led consortia and projects like the Human Brain Project, European institutions provide the necessary theoretical frameworks and device modeling standards that underpin technology robustness, bridging the gap between post-Moore semiconductor values and practical utility. In China, efforts emphasize vertical integration and industrial implementation. Leading enterprises, including China Electronics Corporation (CEC) Haikang, have established dedicated pilot centers [133] to accelerate the translation of laboratory-level neuromorphic concepts into vertically integrated industrial pipelines. The model leverages a vast IoT-driven deployment market to foster a closed-loop ecosystem of device R&D, pilot fabrication, and system evaluation. Meanwhile, East Asia, particularly South Korea, capitalizes on established supply chain advantages and display manufacturing synergies to drive rapid device iteration. The tight collaboration between universities, national laboratories, and industrial leaders (e.g., Samsung) facilitates the integration of ISMC-related platforms into mature semiconductor workflows. Ultimately, transcending a mere technology race, this convergence of global wisdom weaves regional strengths into a unified catalyst, propelling the entire ISMC ecosystem toward a prosperous new era of post-von Neumann intelligence.

**Fig. 17:**
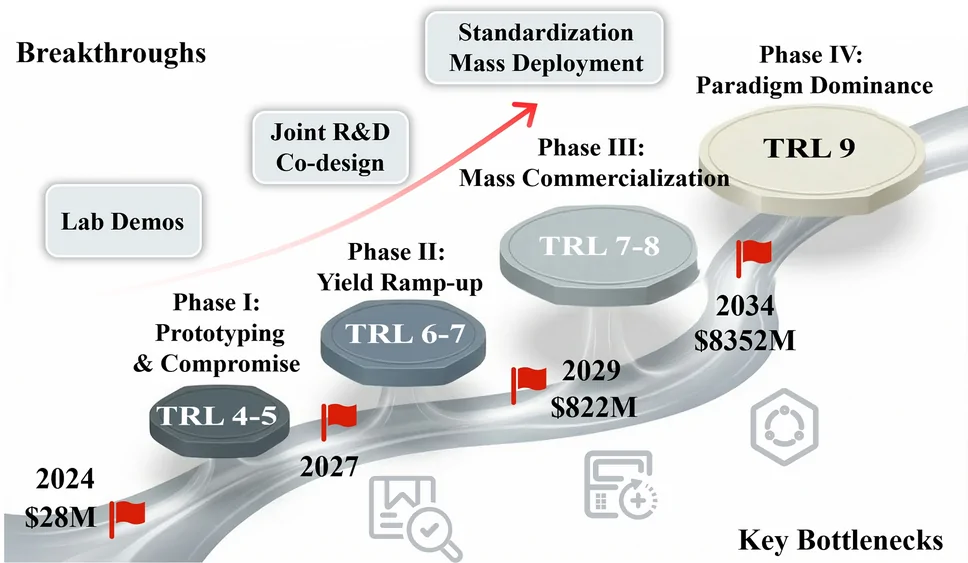
The technology maturity and commercialization roadmap for ISMC from 2024 to beyond 2034. The timeline illustrates the exponential market growth forecasted by Yole Group [135]. TRL, Technology Readiness Level

From a market standpoint, ISMC is rapidly approaching a critical inflection point, poised for exponential growth over the coming decade [134]. According to the forecast by Yole Group [135], the global market for neuromorphic sensing and computing will expand from a nascent $28 million in 2024 to approximately $822 million by 2029, before accelerating dramatically to exceed $8.3 billion by 2034. As illustrated in Fig. 17, the commercialization trajectory of ISMC can be mapped across four distinct phases.

In the short term, the transition toward non-von Neumann architectures is driven by mature digital SRAM and MRAM-based NMC. The highly anticipated fully analog ISMC, powered by emerging NVM-based IMC cores, remains largely at Technology Readiness Level (TRL) 4–5. Its commercialization is fundamentally hindered by severe device non-idealities, such as conductance drift in PCRAM [136] and extreme device-to-device variability in RRAM [137], which severely degrade the computational accuracy and yield of large-scale CBAs. Consequently, early breakthroughs rely heavily on lab-scale prototypes, such as Samsung’s demonstration of an MRAM-based IMC macro [138] and Intel’s research-grade Loihi systems [139].

**Fig. 18:**
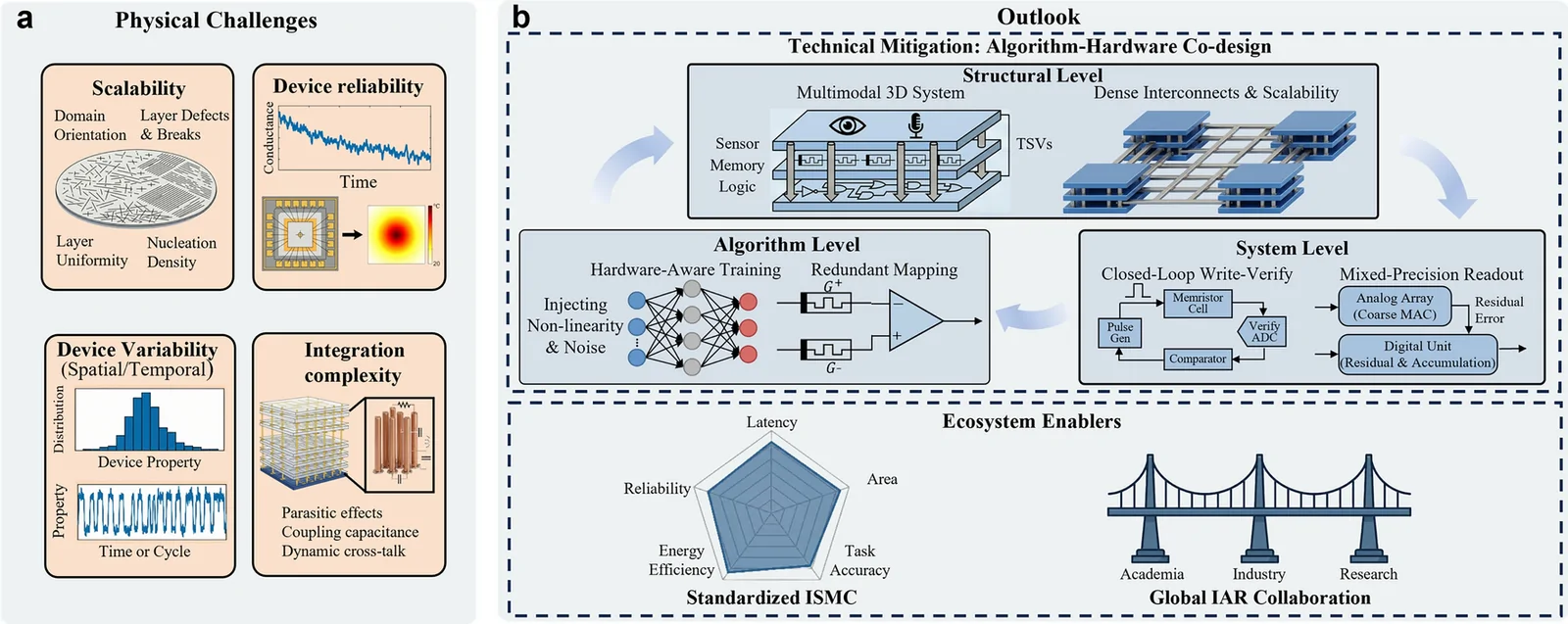
Challenges and future outlook of ISMC systems. **a** Physical Challenges arising from intrinsic device stochasticity, including variability, conductance drift, and thermal noise in memristive arrays. **b** Outlook for overcoming these constraints through Technical Mitigation and Ecosystem Enablers

As analog ISMC progresses toward pilot runs and yield ramp-up (TRL 6–7), the endurance gap emerges as a critical limitation. While traditional SRAM offers nearly unlimited write endurance cycles (> 10^16^), emerging NVMs are typically restricted to 10^5^ to 10^12^ cycles, inherently limiting them to inference-only tasks rather than dynamic online training (Table 1). Bridging this gap necessitates robust algorithm-hardware co-design, such as hardware-aware training algorithms, to computationally compensate for physical degradation. Ultimately, achieving mass commercialization and architectural paradigm dominance (projected post-2034, TRL 9) requires building a mature ecosystem through standardization, such as establishing a universal Neuromorphic Intermediate Representation [140]. By systematically addressing these material and architectural bottlenecks through continuous IAR collaboration, ISMC is well positioned to evolve into a foundational hardware paradigm for pervasive and embodied AI over the next decade (Fig. 18).

## Challenges and Future Outlook

### Challenges

ISMC represents a fundamental shift in how intelligent hardware systems acquire, process, and interpret information. By dissolving the rigid boundaries between sensing, storage, and computation, ISMC addresses long-standing inefficiencies associated with data movement, latency, and energy consumption in conventional architectures. Recent advances in memristive and ferroelectric devices, low-dimensional and multifunctional materials, 3D heterogeneous integration, and neuromorphic architectures collectively demonstrate that the essential building blocks for distributed, in-sensor intelligence are rapidly maturing.

Despite this progress, the transition of ISMC from laboratory demonstrations to scalable, industrial-grade technologies remains contingent on addressing critical cross-layer impediments, most notably the intrinsic stochasticity of analog substrates. Unlike deterministic digital logic, emerging non-volatile memories face severe reliability constraints manifesting as both spatial cell-to-cell variability arising from process mismatch and temporal cycle-to-cycle instability driven by stochastic switching dynamics [141]. Specifically, during read and write operations, analog devices suffer from severe dynamic fluctuations, including thermal noise, random telegraph noise, and programming nonlinearity and asymmetry [142]. Furthermore, long-term reliability is threatened by conductance drift over time and thermal accumulation in dense arrays. For instance, in PCRAM, the structural relaxation of the amorphous phase leads to a spontaneous decrease in conductance [136], necessitating rigorous physical-level management to ensure stability under continuous operation [143]. In analog ISMC arrays, these non-idealities act as intrinsic computational noise that directly distorts analog MAC operations. Since analog MAC relies on precise weight mapping, any spatial or temporal deviations inevitably translate to calculation errors that accumulate across layers, potentially degrading inference accuracy [60].

Beyond device-level stochasticity, realizing industrial-grade ISMC systems is heavily bottlenecked by controllable scalability and exponential integration complexity. From a scalability perspective, expanding emerging functional materials into wafer-level, highly uniform arrays is hindered by current synthesis limitations, particularly in controlling nucleation density, domain orientation, and defect distribution across macroscopic areas. Meanwhile, scaling up these arrays introduces severe macroscopic parasitic effects. In massive crossbar architectures, interconnect resistance leads to substantial IR drops along the bitlines and wordlines, causing devices situated far from the drivers to receive distorted voltages, which further compromises weight mapping precision and MAC fidelity [142]. Furthermore, as arrays transition from 2D planar layouts to 3D vertical stacking to meet extreme computing density demands, manufacturing and architectural complexity grow exponentially. This dense 3D integration introduces severe signal integrity degradation, as parasitic capacitance and resistance from vertical interconnects induce dynamic cross-talk and severe RC delays at high frequencies. Additionally, the sophisticated peripheral circuits required to drive, read, and calibrate these massive arrays risk creating a new “peripheral circuit wall,” where the area and power overhead overshadow the computing core itself [97].

### Outlook

To mitigate these physical constraints, the design paradigm is increasingly shifting from “error avoidance” to “computing with imperfect hardware.” This requires algorithm-hardware co-design frameworks to evolve beyond static mapping strategies.

As the foundational step in co-design, hardware characteristic modeling translates physical non-idealities, such as spatial conductance variance or temporal drift, into parameterized statistical distributions. By embedding these statistical profiles directly into the software training framework, algorithms can explicitly perceive and adapt to specific hardware constraints prior to deployment. Building upon this, training methods incorporating noise injection are essential to generate weight representations that are robust against hardware defects. By integrating empirical device-specific variation models into the forward pass during offline learning, the optimization process is explicitly guided toward flatter minima in the loss landscape. This topological shift in the weight space ensures that the neural network retains robust inference accuracy even when mapped onto highly stochastic analog substrates [144].

Furthermore, bridging the gap between high-level algorithmic instructions and mixed-signal ISMC architectures relies on specialized hardware-aware compilation frameworks. Rather than treating the hardware as an abstract black box, these compilers perform explicit graph lowering and micro-architectural mapping. Specifically, during instruction splitting, the compiler decomposes high-level neural network computational graphs into hardware-executable micro-instructions (e.g., spike generation, synaptic updates, and synchronization) [145]. This involves structurally dividing large weight matrices into smaller sub-matrices to fit constrained physical crossbars [146], and logically separating synaptic integration from neuronal state updates onto dedicated heterogeneous computing units to maximize execution efficiency [147]. Meanwhile, to deploy these decomposed instructions across massive multi-core neuromorphic arrays, the compiler employs sophisticated parallel scheduling strategies and communication optimizations. By constructing inter-processor communication graphs, the compiler establishes transaction orders to resolve data dependencies, effectively minimizing resource contention and overlapping communication with parallel computation [148]. Moreover, these mapping tools optimize the spatial placement of neural clusters to preserve local connectivity and orchestrate asynchronous spike routing over networks-on-chip by merging spike events, significantly reducing traffic density across the mixed-signal boundaries [149].

During online operation, algorithmic intelligence plays a vital role in orchestrating hardware maintenance. The system achieves this by periodically transferring weights from volatile to non-volatile memory via an iterative, closed-loop read–verify–write tuning mechanism. By distributing these column-by-column updates in the background while other layers compute, the architecture effectively hides the transfer time and avoids excessive latency. Ultimately, this orchestrated tuning successfully compensates for major device non-idealities including update asymmetry, CMOS variability, and conductance drift [143]. Furthermore, at the system level, mixed-precision architectures represent a quintessential algorithm-hardware co-design that decouples massive computations from precision-critical updates. Specifically, energy-efficient analog arrays execute massive, low-precision parallel MAC operations, while high-precision digital units compute and iteratively accumulate residual errors. This synergy effectively compensates for inherent analog inaccuracies, thereby achieving software-equivalent training and inference accuracy [150].

Beyond individual device reliability, realizing the full potential of ISMC requires structural advances in scalability and standardization. 3D heterogeneous integration will be decisive in resolving interconnect bottlenecks between sensing and computing layers, enabling dense multimodal processing [151]. Concurrently, the establishment of community-wide benchmarking protocols is critical. Since ISMC performance cannot be captured by isolated metrics like throughput alone, new standards need to encompass device physics, circuit behavior, and task-level accuracy to facilitate fair comparisons between disparate material systems and digital baselines [152]. Looking ahead, sustained progress in ISMC will depend on close coordination across materials science, semiconductor engineering, neuromorphic computing, and application-driven system design. IAR collaboration is poised to play a decisive role in bridging fundamental discoveries with manufacturable platforms, enabling the convergence of new device primitives, co-designed algorithms, and scalable integration technologies. If these challenges are addressed in a systematic and collaborative manner, ISMC has the potential to evolve into a foundational hardware paradigm, enabling autonomous machines, intelligent edge systems, and next-generation cyber-physical platforms that operate efficiently, adaptively, and in close interaction with the physical world.
