# Correction: In-Sensor-Memory Computing for Post-Von Neumann Intelligence: A Perspective

**DOI:** 10.1007/s40820-026-02217-5

**Published:** 2026-05-13

**Authors:** Hongyu Tang, Ninghai Yu, Pengsheng Min, Ruiqian Guo, Guoqi Zhang

**Affiliations:** 1 College of Intelligent Robotics and Advanced Manufacturing, Fudan University, Shanghai, 200433 People’s Republic of China; 2 Shanghai Engineering Technology Research Center for SiC Power Device, Fudan University, Shanghai, 200433 People’s Republic of China; 3 EEMCS Faculty, Delft University of Technology, Delft, 2628CD The Netherlands

**Correction to: Nano-Micro Lett. (2026) 18:338** 10.1007/s40820-026-02191-y

Following publication of the original article [1], the authors reported that the Acknowledgments and Authors’ Contributions sections need to be updated.

The incorrect version is:

**Acknowledgements** Ruiqian Guo contributed to conceptualization, review, and supervision. Guoqi Zhang contributed to review and feedback.

**Author Contributions** Hongyu Tang contributed to investigation, original draft writing, visualization, and funding acquisition. Ninghai Yu and Pengsheng Min were involved in original draft writing, visualization, and review. Ruiqian Guo and Guoqi Zhang contributed to conceptualization, review, and supervision.

The correct version is:

**Acknowledgments** This work was financially supported by the National Natural Science Foundation of China [Grant No. 6250030237], the Shanghai Natural Science Foundation [Grant No. 25ZR1402023], and Shanghai Research Center for Silicon Carbide Power Devices Engineering &Technology Project [Grant No. 19DZ2253400]. We thank Prof. Wenzhong Bao at Fudan University for his valuable comments on this paper.

**Authors’ Contributions** Hongyu Tang: Investigation, original draft writing, visualization, funding acquisition. Ninghai Yu and Pengsheng Min: original draft writing, visualization, review. Ruiqian Guo: Conceptualization, review, supervision. Guoqi Zhang: review and feedback.

The original article [1] has been corrected.

## References

[1] H. Tang, N. Yu, P. Min et al., In-sensor-memory computing for post-Von Neumann intelligence: a perspective. Nano-Micro Lett. **18**, 338 (2026). 10.1007/s40820-026-02191-y 10.1007/s40820-026-02191-y PMC13092463 42002681

